# IRF4 Regulates the Ratio of T-Bet to Eomesodermin in CD8^+^ T Cells Responding to Persistent LCMV Infection

**DOI:** 10.1371/journal.pone.0144826

**Published:** 2015-12-29

**Authors:** Ribhu Nayar, Elizabeth Schutten, Sonal Jangalwe, Philip A. Durost, Laurie L. Kenney, James M. Conley, Keith Daniels, Michael A. Brehm, Raymond M. Welsh, Leslie J. Berg

**Affiliations:** 1 Department of Pathology, University of Massachusetts Medical School, Worcester, MA 01605, United States of America; 2 Program in Molecular Medicine, University of Massachusetts Medical School, Worcester, MA 01605, United States of America; Indiana University, UNITED STATES

## Abstract

CD8^+^ T cell exhaustion commonly occurs in chronic infections and cancers. During T cell exhaustion there is a progressive and hierarchical loss of effector cytokine production, up-regulation of inhibitory co-stimulatory molecules, and eventual deletion of antigen specific cells by apoptosis. A key factor that regulates T cell exhaustion is persistent TCR stimulation. Loss of this interaction results in restoration of CD8^+^ T cell effector functions in previously exhausted CD8^+^ T cells. TCR stimulation is also important for the differentiation of Eomes^hi^ anti-viral CD8^+^ effector T cells from T-bet^hi^ precursors, both of which are required for optimal viral control. However, the molecular mechanisms regulating the differentiation of these two cell subsets and the relative ratios required for viral clearance have not been described. We show that TCR signal strength regulates the relative expression of T-bet and Eomes in antigen-specific CD8^+^ T cells by modulating levels of IRF4. Reduced IRF4 expression results in skewing of this ratio in the favor of Eomes, leading to lower proportions and numbers of T-bet^+^ Eomes^-^ precursors and poor control of LCMV-clone 13 infection. Manipulation of this ratio in the favor of T-bet restores the differentiation of T-bet^+^ Eomes^-^ precursors and the protective balance of T-bet to Eomes required for efficient viral control. These data highlight a critical role for IRF4 in regulating protective anti-viral CD8^+^ T cell responses by ensuring a balanced ratio of T-bet to Eomes, leading to the ultimate control of this chronic viral infection.

## Introduction

Acute virus infections are characterized by the formation of robust CD8^+^ T cell effector responses followed by the generation of immunological memory. Both CD8^+^ effector T cells as well as CD8^+^ memory cells produce a variety of cytokines and cytotoxic molecules, and have high proliferative capacity [1]. In contrast, during chronic viral infections, high viral loads cause CD8^+^ T cell exhaustion that is characterized by hierarchal loss of effector functions and eventual deletion of antigen-specific cells [2–4]. The remaining virus-specific CD8^+^ T cells lose the ability to make IFNγ, TNFα, and IL-2, and up-regulate high levels of inhibitory receptors such as PD-1 and LAG-3. Eventually the cells become completely dysfunctional and are deleted by apoptosis [2]. T cell exhaustion was initially thought to be a viral immune evasion mechanism, but recent studies have indicated that it serves to protect the host from T cell-mediated immunopathology [5,6].

Many factors regulate T cell exhaustion. The expression of the immuno-suppressive cytokine IL-10 and inhibitory co-receptors like PD-1 enhance T cell exhaustion, whereas help from CD4^+^ T cells aids in the restoration of CD8^+^ T cell function [7–10]. Persistent T cell signaling due to high viral loads and increased MHC-I presentation is detrimental as well as beneficial during chronic infection. Increased antigen presentation results in reduced numbers and impaired function of anti-viral CD8^+^ T cells; however, loss of this interaction also leads to poor viral control [4]. Antigen is also required for the long-term maintenance of virus-specific cells during chronic infections, as these cells do not undergo homeostatic proliferation in response to IL-7 and IL-15; instead, they require viral antigen [11,12].

In the presence of a persistent infection, exhausted CD8^+^ T cells were found as two distinct subsets, one subset expressing high levels of the transcription factor, T-bet, and the other subset expressing high levels of the related transcription factor, Eomesodermin (Eomes). Further, Paley, *et*.*al*. showed that, in response to antigenic stimulation, T-bet^hi^ cells give rise to Eomes^hi^ cells. T-bet^hi^ cells were found to be less exhausted and exhibited higher proliferative rates in response to antigen, while the Eomes^hi^ cells were more exhausted, but exhibited greater cytotoxic activity than the T-bet^hi^ cells. Both subsets were essential for viral control, as loss of either transcription factor resulted in viral persistence [13]. These data indicated that optimal expression of T-bet and Eomes, and the presence of both CD8^+^ T cell subsets, were essential for efficient viral control. However, the pathway that regulates the differentiation of antigen-specific T cells into these subsets, and whether a specific ratio of the two subsets is critical for viral control, are not known.

IRF4 is a pleiotropic transcription factor that regulates a myriad of functions in a wide variety of cell populations [14]. Recently, we and others have shown that levels of IRF4 in CD8^+^ T cells are regulated by the strength of TCR signaling. Thus, robust expression of IRF4 is important for maximal CD8^+^ T cell expansion in response to acute viral infections [15–19]. When IRF4 levels are reduced by a haplo-deficiency of the *Irf4* gene, the magnitude of the CD8^+^ T cell response is dramatically impaired. The decreased numbers of virus-specific T cells are accounted for by a reduction in terminal effector cells (SLEC; KLRG1^hi^CD127^lo^) without a significant effect on the numbers of memory precursor effector cells (MPEC, KLRG1^lo^CD127^hi^) [15]. These studies also highlighted a role for IRF4 in the expression of key transcription factors T-bet and Eomes, important for differentiation and maintenance of SLEC and MPEC populations, respectively, during acute infections [15–21].

Here we show that TCR signal strength maintains an optimal balance of T-bet to Eomes, and that this process is regulated by the levels of IRF4 expressed. Reduced expression of IRF4 skews this ratio in the favor of Eomes during infection with LCMV-clone 13, resulting in reduced differentiation of T-bet^+^ Eomes^-^ precursors and impaired viral control. Reducing Eomes expression in *Irf4* heterozygous mice re-establishes the protective balance of T-bet to Eomes, restores differentiation of T-bet^+^ Eomes^-^ precursors, and ultimately rescues defective viral clearance. These data indicate a critical role for IRF4 in regulating T cell exhaustion by balancing the relative expression of T-bet and Eomes during chronic infection. Overall, these findings demonstrate that reduced differentiation of the T-bet^+^ Eomes^-^ CD8^+^ T cell population impairs viral clearance, whereas a partial reduction in Eomes expression can restore viral control during persistent LCMV-clone 13 infections.

## Results

### TCR signal strength via IRF4 regulates the ratio of T-bet and Eomesodermin in activated CD8^+^ T cells

**Fig 1:**
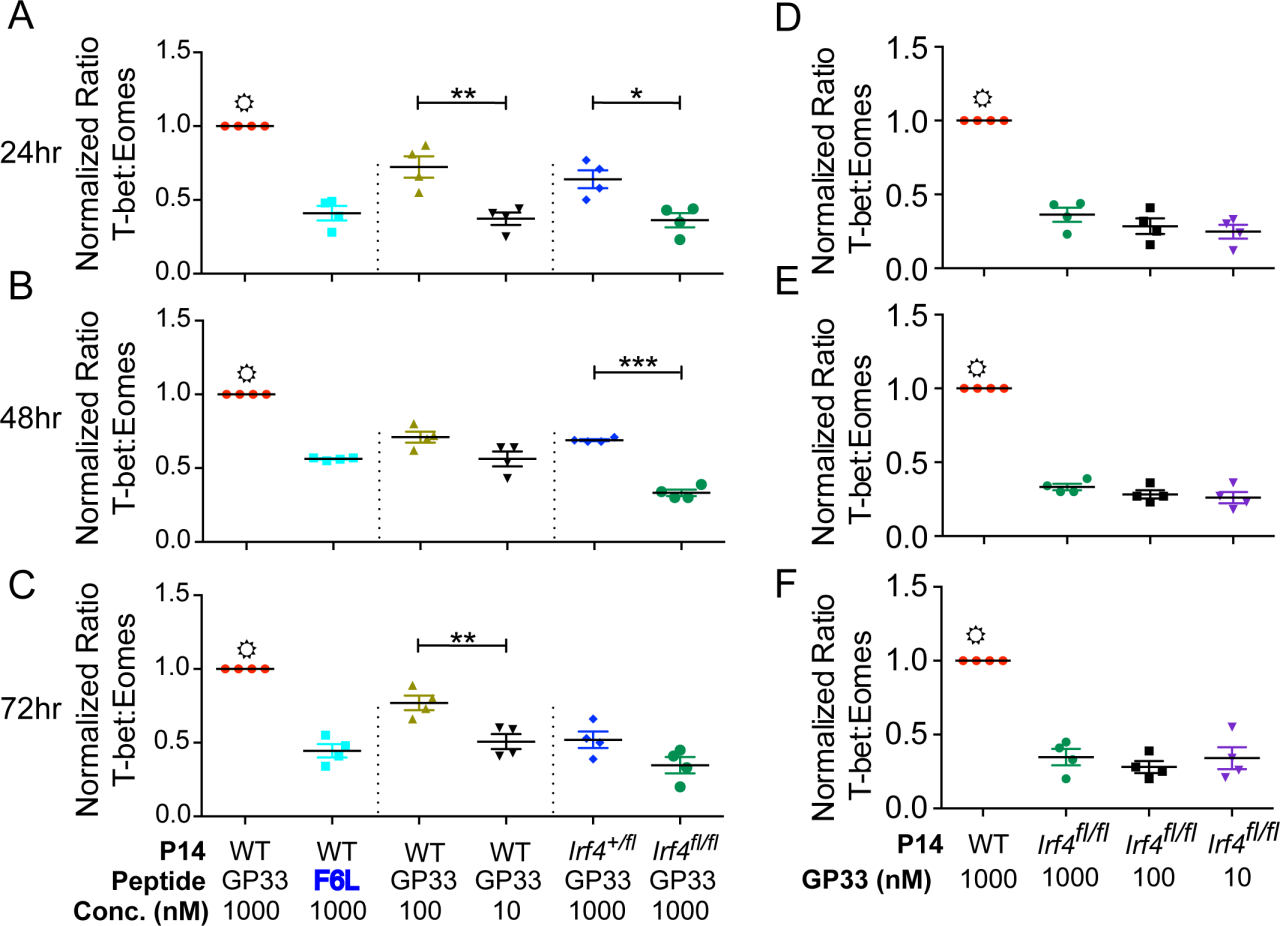
IRF4 regulates the T-bet to Eomesodermin ratio in stimulated CD8^+^ T cells. P14 WT, P14 *Irf4* ^*+/fl*^ or P14 *Irf4* ^*fl/fl*^ cells were stimulated with the indicated concentrations of GP33 and F6L peptides *in-vitro*. At (A, D) 24, (B, E) 48, or (C, F) 72 h, cells were stained and analyzed for T-bet and Eomes expression. The graphs show compilations of the ratios of MFIs for T-bet relative to Eomes, each normalized to the value for 1μM GP33-stimulated cells at each time-point. Data were generated from gated live CD8^+^CD45.2^+^CD44^hi^ T cells analyzed in four independent experiments. ☼, denotes statistically significant difference between 1μM GP33-stimulated cells and all other sample groups on the graph as determined by unpaired t test with Welch’s correction.

To understand the role of TCR signal strength in the differentiation of CD8^+^ T cell subsets, we utilized P14 TCR transgenic TCRα^-/-^ cells (hereafter referred to as P14 WT), that respond to the lymphocytic choriomeningitis virus (LCMV) GP_33-41_ epitope (GP33) bound to H2-D^b^; additionally, P14 WT cells respond to a lower affinity ‘F6L’ peptide variant of GP_33-41_, containing a single amino acid substitution from phenylalanine to leucine at the sixth position of the peptide. This alteration leads to a 5-fold decrease in the affinity of the P14 TCR for the F6L-H2-D^b^ complex relative to GP_33-41_- H2-D^b^, and as a consequence, a 100-1000-fold reduction in functional avidity [22]. P14 WT T cells were stimulated *in vitro* and then examined for T-bet (S1A Fig) and Eomes (previously published in [15]) expression by intracellular staining. At 24hr post-stimulation, P14 WT T cells stimulated with the high affinity GP33 epitope, expressed a higher T-bet to Eomes ratio relative to cells stimulated with the lower affinity F6L epitope (Fig 1A). A similar trend was also observed when P14 cells were stimulated with decreasing doses of the GP33 peptide. These data indicate that variations in TCR signal strength, such as those achieved by varying the affinity of the peptide-MHC for the TCR or by varying the dose of stimulating peptide, affect the relative expression levels of these key transcription factors.

The transcription factor IRF4 is upregulated in CD8^+^ T cells in a graded manner in response to variations in TCR signal strength. Furthermore, IRF4 is known to be a negative regulator of Eomes and a positive regulator of T-bet expression in CD8^+^ T cells [15–18,23,24]. To determine if alterations in IRF4 expression affect the relative up-regulation of T-bet and Eomes, we compared P14 WT T cells to P14 TCRα^-/-^ cells lacking one or two functional alleles of *Irf4* (*Irf4* ^*+/fl*^ *X CD4-Cre* and *Irf4* ^*fl/fl*^ *X CD4-Cre*; referred to as P14 *Irf4* ^*+/fl*^ and P14 *Irf4* ^*fl/fl*^, respectively). In response to stimulation with the GP33 peptide, P14 WT cells expressed the highest T-bet to Eomes ratio, while P14 *Irf4* ^*+/fl*^ cells showed a reduced T-bet to Eomes ratio, and P14 *Irf4* ^*fl/fl*^ cells exhibited the lowest T-bet to Eomes ratio (Fig 1A). This pattern was observed at all time points examined (Fig 1B and 1C). Furthermore, no alterations in the ratio of T-bet to Eomes expression was observed when P14 *Irf4* ^*fl/fl*^ cells were stimulated with varying doses of GP33 peptide (Fig 1D–1F). These data indicate that the strength of TCR signaling via IRF4 regulates the relative expression of T-bet and Eomes in CD8^+^ T cells.

### Levels of IRF4 regulate CD8^+^ T cell differentiation into T-bet^+^ Eomes^-^ and T-bet^-^ Eomes^+^ subsets in response to LCMV Cl13 infection

The data described thus far indicated a critical role of IRF4 in maintaining the balance of T-bet and Eomes *in-vitro*. To determine whether the levels of IRF4 regulate the relative expression of T-bet and Eomes *in-vivo*, WT, *Irf4* ^*+/fl*^ *X CD4-Cre* and *Irf4* ^*fl/fl*^ *X CD4-Cre* mice (henceforth referred to as *Irf4* ^*+/fl*^ and *Irf4* ^*fl/fl*^, respectively) were infected with an exhausting dose of LCMV-clone 13. Due to the defects in regulatory T cell populations in *Irf4* ^*fl/fl*^ mice, CD4^+^ and CD8^+^ T cell populations are activated in *Irf4* ^*fl/fl*^ mice even in the absence of infection [23,25]. As a result, we focused on understanding how modest reductions, rather than a complete loss of IRF4 expression, impacted the protective response to persistent virus infection.

**Fig 2:**
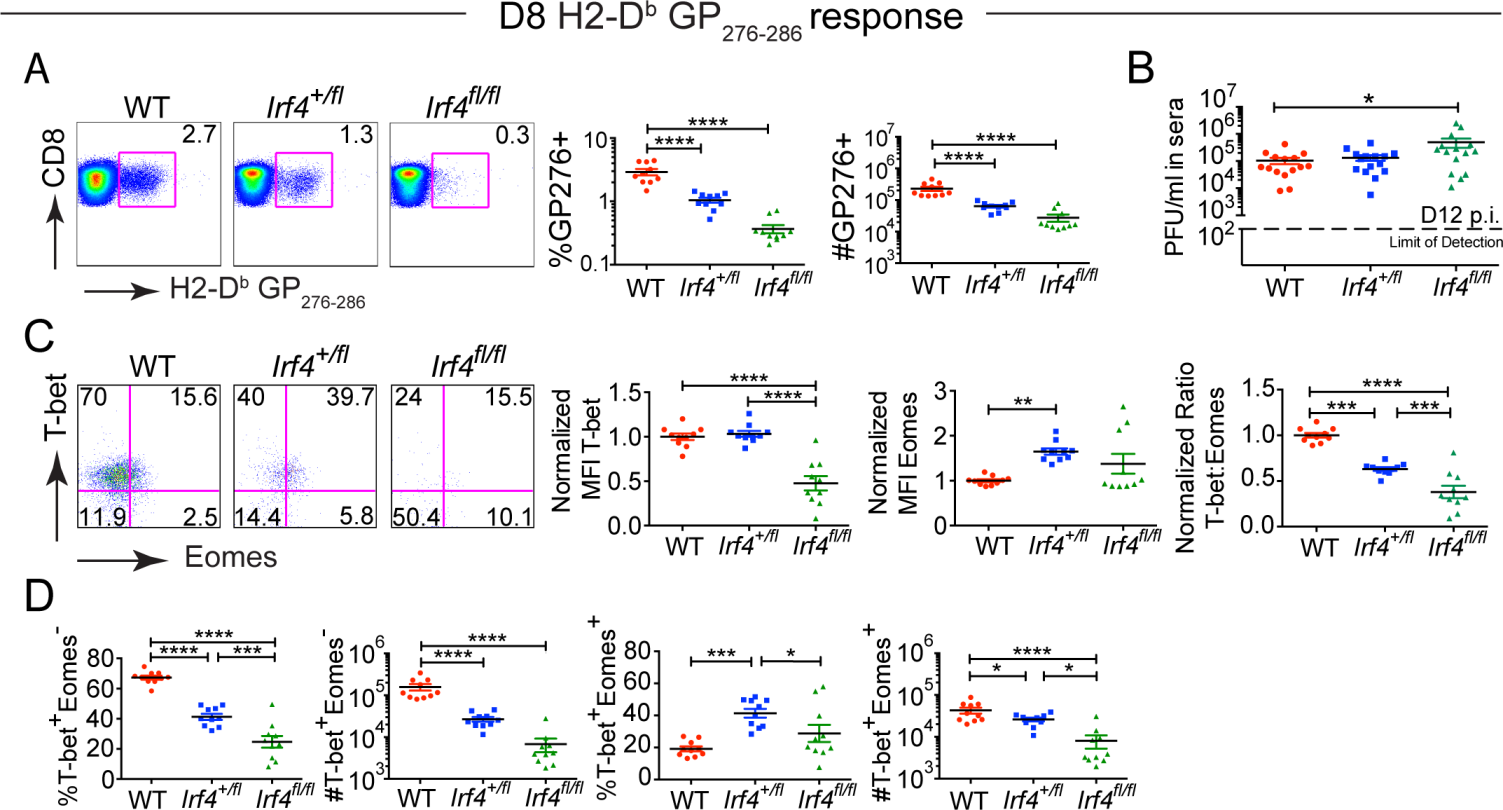
*Irf4* gene dosage regulates CD8^+^ T cell clonal expansion in response to LCMV-clone 13 infection and the differentiation of T-bet^hi^ and Eomes^hi^ subsets. (A) Splenocytes from LCMV-clone 13-infected WT, *Irf4* ^*+/fl*^ and *Irf4* ^*fl/fl*^ mice were harvested at D8 p.i. and stained with a viability dye, LCMV-specific H2-D^b^-GP276 tetramer, and antibodies to CD8, T-bet and Eomes. Dot plots show CD8 versus H2-D^b^-GP276 tetramer staining (left). Graphs show compilations of proportions and numbers from D8 p.i. (right). Each data point represents an individual mouse and data are a compilation of three independent experiments. (B) LCMV-clone 13 titers in serum at D12 post-infection. Dotted line indicates limit of detection. Each data point represents an individual mouse and data are a compilation of four independent experiments. (C) Representative dot plots show T-bet vs Eomes staining on gated CD8^+^ live H2-D^b^-GP276 specific cells at D8 p.i (left). Graphs show the MFI of T-bet and Eomes, each normalized to the average of WT samples for live CD8^+^ H2-D^b^-GP276 specific cells, and the ratio of normalized MFIs for T-bet relative to Eomes (right). Each data point represents an individual mouse and data are a compilation of three independent experiments. (D) Graphs show compilations of proportions and numbers of T-bet^+^ Eomes^-^ and T-bet^+^ Eomes^+^ cells. Each data point represents an individual mouse and data are a compilation of three independent experiments. Significant differences determined by Ordinary one-way ANOVA using Tukey’s multiple comparison test.

Virus specific responses to H2-D^b^/GP_276–286_ and H2-D^b^/GP_33–41_ were examined using MHC-peptide tetramers. At D8 post-infection, *Irf4* ^*+/fl*^ and *Irf4* ^*fl/fl*^ mice had reduced proportions and numbers of virus-specific CD8^+^ T cells compared to WT mice, consistent with previous studies of acute virus infections in these mice (Fig 2A and S2A Fig) [15–19]). At D12 post-infection, WT and *Irf4* ^*+/fl*^ had similar levels of virus in their sera, whereas *Irf4* ^*fl/fl*^ mice showed slightly reduced control of the virus infection (Fig 2B). Analysis of T-bet and Eomes expression in the virus-specific CD8^+^ T cells indicated that WT cells had the highest T-bet to Eomes ratio, followed by *Irf4* ^*+/fl*^ CD8^+^ T cells, and then *Irf4* ^*fl/fl*^ cells (Fig 2C and S2B Fig).

In response to LCMV-clone 13 infection, anti-viral CD8^+^ T cells differentiate into T-bet^+^ Eomes^-^ precursors that give rise to T-bet^-^ Eomes^+^ progeny in response to antigenic stimulation [13]. Examination of these subsets at D8 p.i. indicated an IRF4 dose-dependent defect in the differentiation of these populations. *Irf4* ^*+/fl*^ mice exhibited a significant decrease in the numbers and proportions of the T-bet^+^ Eomes^-^ precursors with a concomitant increase in the differentiation to T-bet^+^ Eomes^+^ cells (Fig 2D and S2C Fig). These double positive cells could potentially be an intermediate population that differentiates into T-bet^-^ Eomes^+^ cells later during infection. As the viral titers are not different between WT and *Irf4* ^*+/fl*^ at D12 p.i. (Fig 2B), these data indicate an inherent defect in the differentiation of CD8^+^ T cells expressing lower levels of IRF4. The differentiation of the *Irf4* ^*fl/fl*^ cells into T-bet^+^ Eomes^-^ and T-bet^+^ Eomes^+^ populations was severely compromised, likely accounting for the increased viral titers in the *Irf4* ^*fl/fl*^ mice. These data indicate that IRF4 regulates the relative expression of T-bet and Eomes and therefore may impact the differentiation of T-bet^+^ Eomes^-^ precursors into T-bet^-^ Eomes^+^ progeny early during persistent LCMV infection. Further, high levels of IRF4 are required for robust expansion of virus-specific T-bet^+^ Eomes^-^ effector CD8^+^ T cells and viral control at early times post-infection.

### WT levels of IRF4 are required to maintain the balance of T-bet to Eomesodermin expression during persistent infection

**Fig 3:**
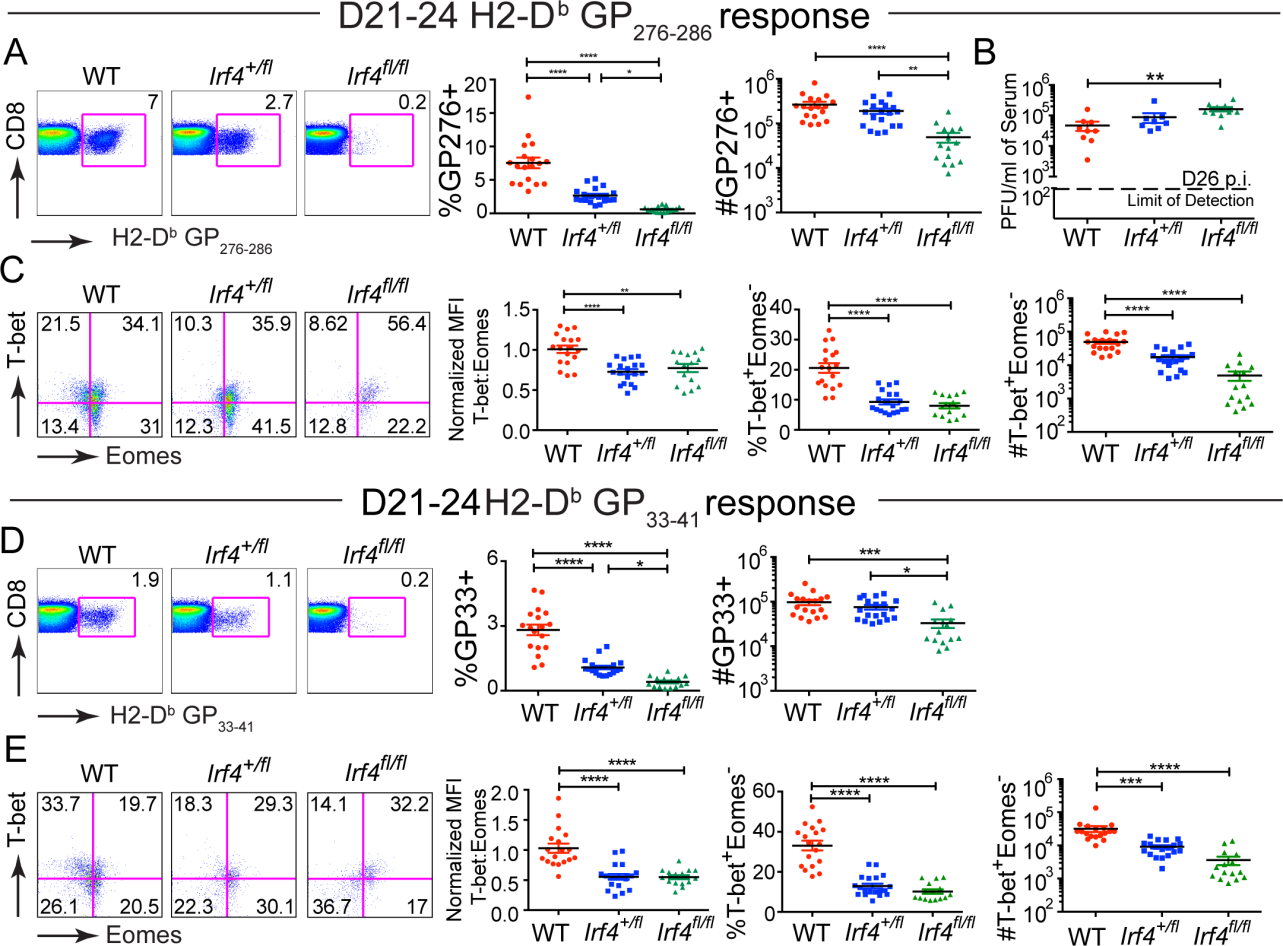
Persistent reduction in virus-specific T-bet^+^ Eomes^-^ CD8^+^ T cells in LCMV-clone 13-infected *Irf4* ^*+/fl*^ mice. (A, D) Splenocytes from LCMV-clone 13-infected WT, *Irf4* ^*+/fl*^ and *Irf4* ^*fl/fl*^ mice were were harvested at D21-24 p.i. and stained with a viability dye, LCMV-specific H2-D^b^-GP276 and H2D^b^-GP33 tetramers, and antibodies to CD8, T-bet and Eomes. Dot plots show CD8 versus H2-D^b^-GP276 (A) or H2-D^b^-GP33 (D) tetramer staining (left). Graphs show compilations of proportions and numbers from D21-24 p.i. (right). Each data point represents an individual mouse and data are a compilation of five independent experiments. (B) LCMV-clone 13 titers in serum at D26 post-infection. Dotted line indicates limit of detection. Each data point represents an individual mouse and data are a compilation of two independent experiments. (C, E) Dot plots show T-bet vs Eomes staining on live CD8^+^ H2-D^b^-GP276 (C) or H2-D^b^-GP33 (E) tetramer positive cells (left). Graph shows the ratio of MFIs of T-bet relative to Eomes, each normalized to the average value of WT samples (middle). Graphs show a compilation of proportions and numbers of T-bet^+^ Eomes^-^ cells for each population (right). Each data point represents an individual mouse and data are a compilation of five independent experiments. Significant differences determined by Ordinary one-way ANOVA using Tukey’s multiple comparison test.

In activated T cells, the expression of IRF4 is regulated by the strength of TCR signaling via the mTOR pathway; furthermore, mTOR signaling is suppressed during chronic infection by signaling through the inhibitory receptor PD-1 [15–17,23,26]. To test the importance of IRF4 expression levels at later timepoints during the persistent infection, we examined CD8^+^ T cell responses to LCMV-clone 13 at D21-24 p.i. At this time point, we observed reduced proportions of virus-specific T cells in the spleens of *Irf4* ^*+/fl*^ mice compared to WT mice; however, absolute numbers were not significantly different (Fig 3A and 3D). As expected, *Irf4* ^*fl/fl*^ mice had significant reductions in both the proportions and numbers of both subsets of virus-specific CD8^+^ T cells.

Examination of T-bet and Eomes expression in virus-specific CD8^+^ T cells at D21-24 p.i. with LCMV-clone 13 indicated that for both of the LCMV epitopes examined, virus-specific *Irf4* ^*+/fl*^ CD8^+^ T cells exhibited an altered T-bet to Eomes ratio relative to WT cells (Fig 3C and 3E). This skewed ratio resulted in reduced differentiation of T-bet^+^ Eomes^-^ and increased differentiation of T-bet^-^ Eomes^+^ populations in *Irf4* ^*+/fl*^ mice relative to WT mice (Fig 3C and 3E and S3A and S3C Fig). High viral titers have been implicated in the excessive proliferation and eventual conversion of the T-bet^hi^ CD8^+^ T cell population into Eomes^hi^ CD8^+^ T cell population in patients with chronic HCV infection [13,27]. However, at day 26 p.i., we observed no difference in serum virus titers between *Irf4* ^*+/fl*^ and WT mice, indicating that the altered CD8^+^ T cell populations were not simply a reflection of differences in viral load (Fig 3B). These data indicate that a modest reduction in IRF4 expression leads to impaired generation of T-bet^+^ Eomes^-^ virus-specific CD8^+^ T cells at later timepoints of the persistent infection.

### Intrinsic role of IRF4 in regulating the balance of T-bet to Eomesodermin expression in CD8^+^ T cells responding to LCMV-clone 13 infection

**Fig 4:**
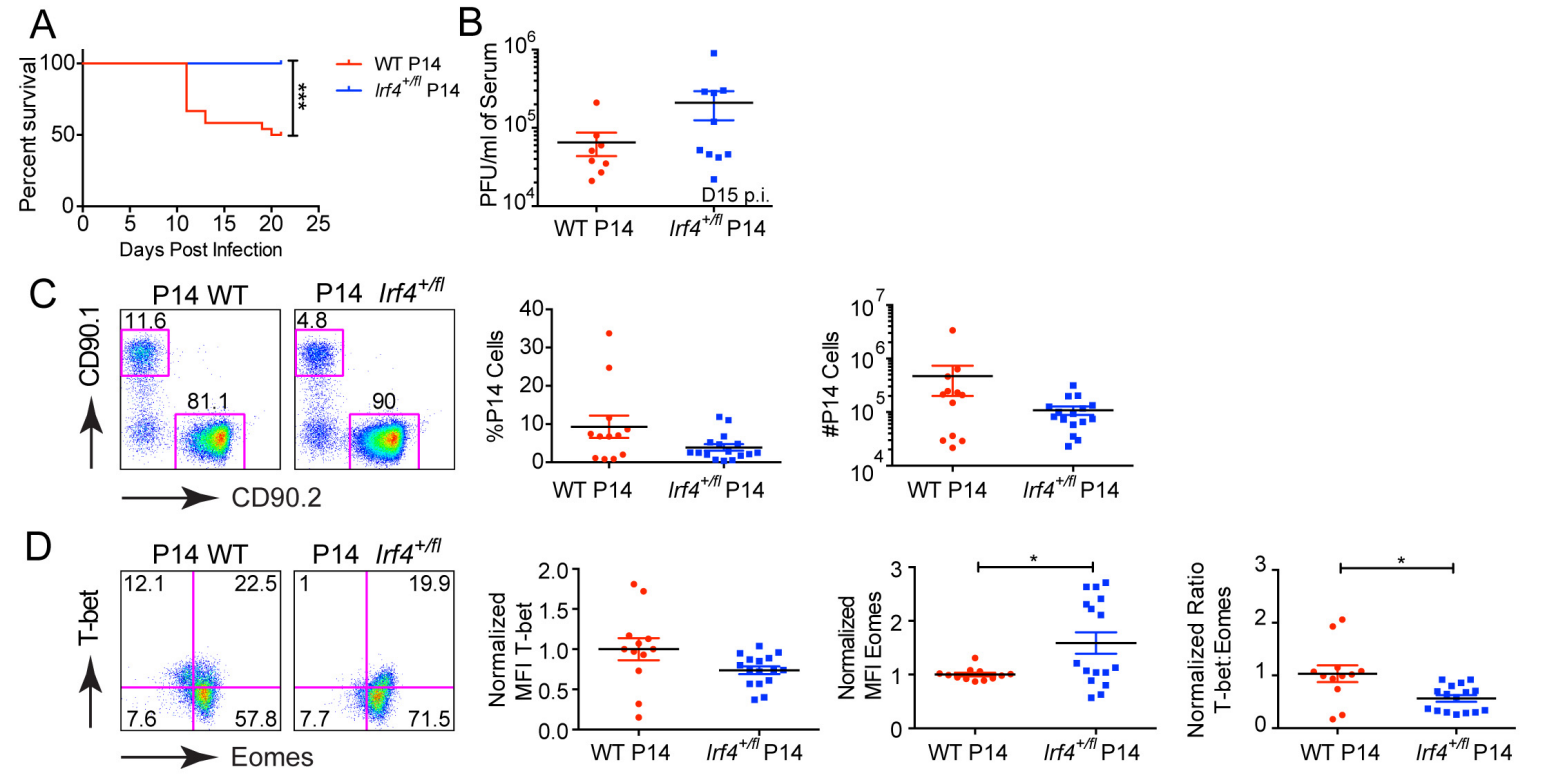
Cell-intrinsic role of IRF4 in regulating the balance of T-bet to Eomesodermin expression in CD8^+^ T cells responding to LCMV-clone 13 infection. 10^4^ CD90.1 WT P14 cells or CD90.1 *Irf4* ^*+/fl*^ P14 cells were transferred into CD90.2 CD4-Cre^+^ host mice and one day later recipients were infected with LCMV-clone 13. (A) Survival curve showing % survival of recipient mice that received either WT or *Irf4* ^*+/fl*^ P14 cells over 22 days. Data are a compilation of three independent experiments. (B) Serum was harvested from recipient mice at D15 post infection and virus titers were determined by plaque assay. Each data point represents an individual mouse and data are a compilation of two independent experiments. (C) Dot plots show CD90.1 (transferred cells) versus CD90.2 (host cells) staining on gated live CD8^+^ T cells (left). Graphs show compilations of proportions and numbers of transferred P14 cells at D22 post-infection (right). Each data point represents an individual mouse and data are a compilation of three independent experiments. (D) Dot plots show T-bet versus Eomes staining on gated live CD90.1^+^ CD8^+^ T cells (left). Graphs show the normalized MFI of T-bet and Eomes, each normalized to the average of P14 WT transferred samples and the ratio of the normalized MFIs for T-bet relative to Eomes, (right). Each data point represents an individual mouse and data are a compilation of three independent experiments. Statistical analysis was determined by Log-rank (Mantel-Cox) test (A) or unpaired t test with Welch’s correction (B-D).

To address whether differences in IRF4 expression were regulating the balance of T-bet to Eomesodermin by acting in virus-specific CD8^+^ T cells, we performed adoptive transfer experiments. P14 WT or P14 *Irf4* ^*+/fl*^ CD8^+^ T cells were transferred into congenic hosts, and recipients were infected with LCMV-clone 13. Beginning at D10 p.i., we observed that a proportion of recipients receiving WT P14 cells were succumbing to the infection, a response not seen in recipients that received P14 *Irf4* ^*+/fl*^ cells (Fig 4A). Analysis of viral titers in the sera of the surviving mice at D15 p.i. indicated that 75% of recipients receiving WT P14 cells had viral titers <7.5x10^4^, whereas for recipients receiving P14 *Irf4* ^*+/fl*^ cells, this value was 2.9x10^5^; however, the means of the two datasets were not significantly different. Based on previous studies showing that fatality from LCMV-cl13 infection results from excessive T cell responses leading to immunopathology [6], these data suggest a more robust effector response of WT compared to *Irf4* ^*+/fl*^ P14 cells to the LCMV-clone 13 infection, consistent with previous observations [19].

To examine the P14 cells directly, surviving mice were analyzed at D22 p.i.. At this timepoint, we recovered fewer P14 *Irf4* ^*+/fl*^ cells from recipient mice compared to WT P14 cells (Fig 4C). Examination of these cells for T-bet and Eomes expression showed that WT P14 cells had increased levels of T-bet and reduced levels of Eomes relative to P14 *Irf4* ^*+/fl*^ cells, resulting in higher T-bet to Eomes ratio in the WT P14 cells (Fig 4D). Overall, these data indicate that the magnitude of IRF4 expression in virus-specific CD8^+^ T cells regulates their expansion, as well as the relative expression of T-bet and Eomes in response to LCMV-clone 13.

### WT levels of IRF4 are essential for optimal control of persistent virus infection

**Fig 5:**
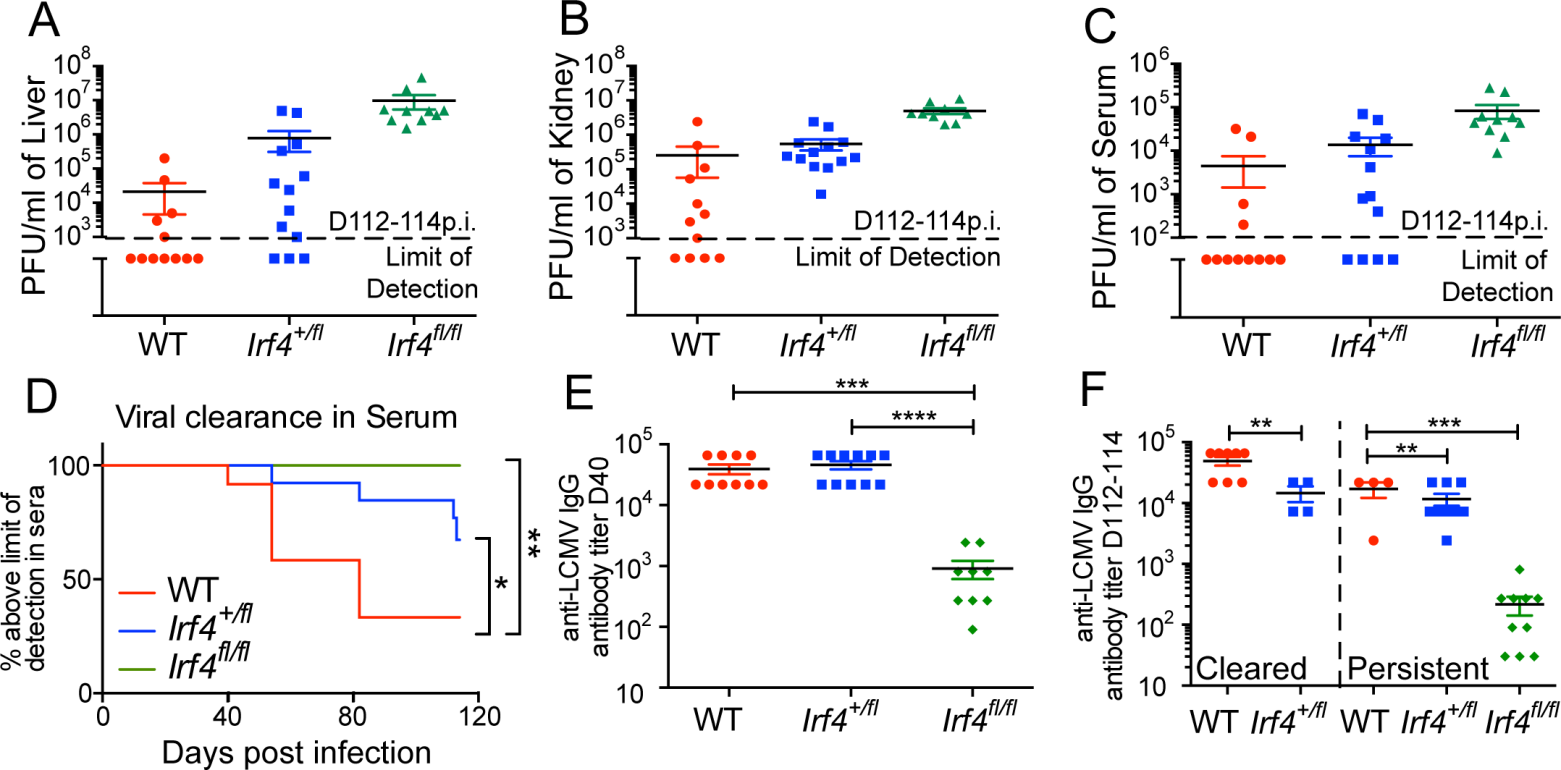
High expression of IRF4 is essential for long-term control of LCMV-clone 13. Kidney (A), livers (B) and sera (C) were harvested from LCMV-clone 13 infected WT, *Irf4* ^*+/fl*^ and *Irf4* ^*fl/fl*^ mice between D112-114 post-infection and virus titers were determined by plaque assay. Dotted line indicates the limit of detection. Each data point represents an individual mouse and data are a compilation of three independent experiments. (D) Serum was harvested from infected mice at various timepoints post-infection. Graph indicates the proportion of mice with viral titers above the limit of detection over time. Data are a compilation of three independent experiments; significant differences were determined by Log-rank (Mantel-Cox) test. (E) Anti-LCMV IgG antibody titers in sera at D40 p.i. Each data point represents an individual mouse and data are a compilation of three independent experiments; significant differences determined by Ordinary one-way ANOVA using Tukey’s multiple comparison test. (F) Anti-LCMV IgG antibody titers in sera at D112-114 p.i. Data are segregated based on serum viral titers; at left are mice with undetectable virus in their sera (cleared) and at right are mice with persistent serum virus titers (persistent). Each data point represents an individual mouse and data are a compilation of three independent experiments; significant differences determined by unpaired t test with Welch’s correction (cleared) and Ordinary one-way ANOVA using Tukey’s multiple comparison test (persistent).

While modest reductions in the levels of IRF4 were inconsequential for early control of virus replication through d26 p.i., long-term studies revealed that *Irf4* ^*+/fl*^ mice had a defect in controlling the persistent virus infection. Relative to WT mice, a lower proportion of *Irf4* ^*+/fl*^ mice controlled LCMV-clone 13 infection in the kidney, liver and serum when examined more than 100 days p.i. (Fig 5A–5C). In addition, enumeration of viral titers in sera over time indicated that the kinetics of viral control were slower for *Irf4* ^*+/fl*^ mice than WT mice (Fig 5D). As expected, *Irf4* ^*fl/fl*^ mice exhibited the highest viral titers and a complete impairment in viral control (Fig 5A–5D).

IRF4 is also important for the differentiation of CD4^+^ Tfh cells that regulate plasma cell differentiation. As previous studies have shown that during LCMV infection reduced antibody responses lead to virus persistence [28,29], we considered whether the slower kinetics of virus control in *Irf4* ^*+/fl*^ mice could be accounted for by reduced anti-LCMV antibody titers. Analysis of LCMV-specific IgG antibody titers at D40 p.i. indicated that *Irf4* ^*fl/fl*^ mice were impaired in antibody production, consistent with the requirement for IRF4 in Tfh differentiation (Fig 5E). However, we observed no difference in antibody titers between WT and *Irf4* ^+/fl^ mice, supporting a CD8^+^ T cell-intrinsic defect in viral control in *Irf4* ^+/fl^ mice. We then analyzed virus-specific antibody titers at D112-114 p.i., a timepoint at which the majority of WT mice were controlling the infection. When antibody data were segregated so that WT and *Irf4* ^*+/fl*^ mice were compared based on whether they had detectable virus in their sera (Fig 5C), we found slightly reduced antibody titers in *Irf4* ^*+/fl*^ mice that were controlling the virus relative to WT mice in this group (Fig 5F). However, no difference in antibody titers between the two genotypes of mice were seen for those that still had persistent virus replication in their sera. These data indicate that high levels of IRF4 expression are essential for optimal long-term control of the persistent LCMV-clone 13 infection, and that differences in viral control are not correlated with differences in anti-viral antibody titers.

**Fig 6:**
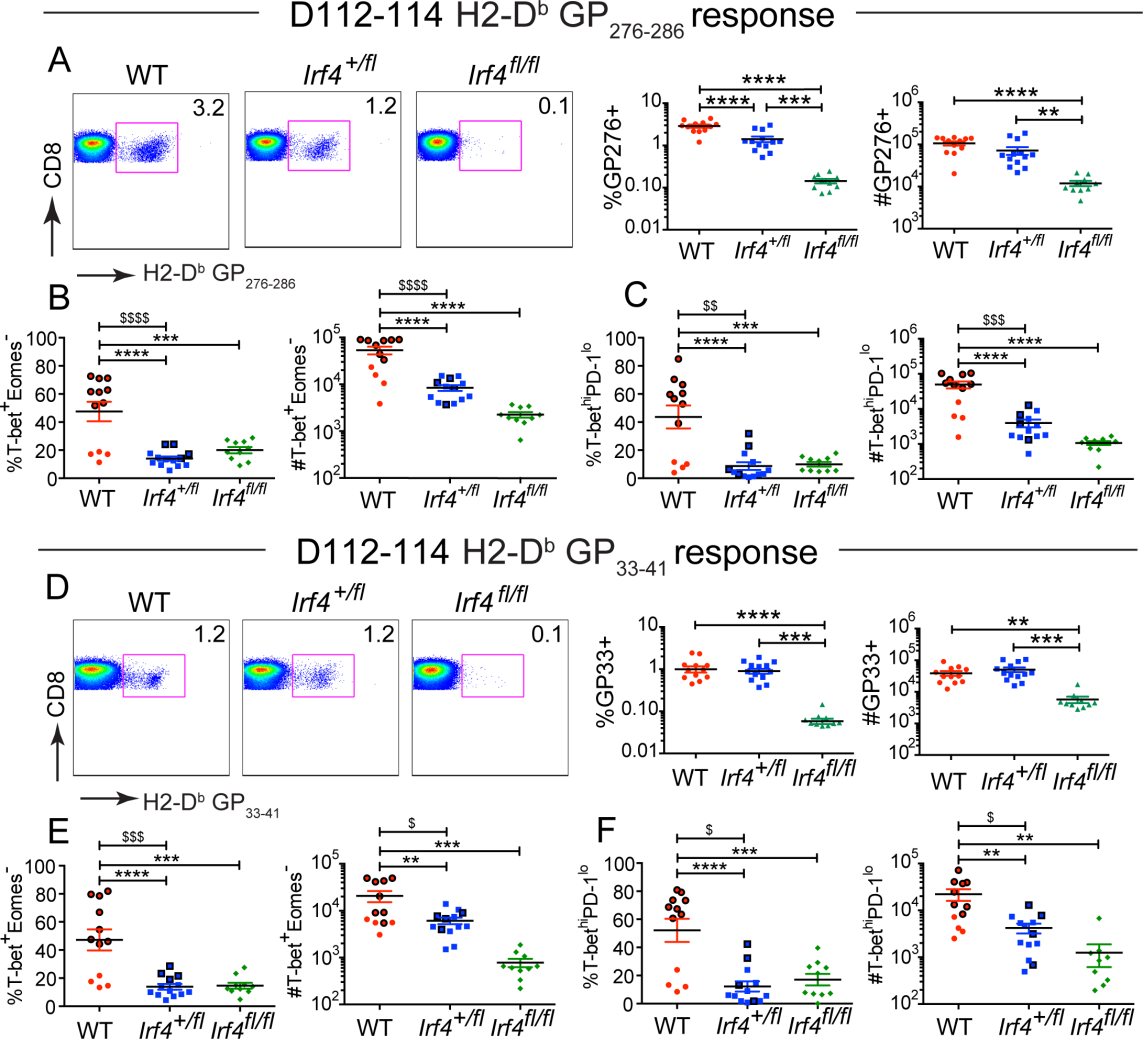
The proportions of T-bet^+^ Eomes^-^ cells correlate with viral control at late timepoints of LCMV-clone 13 infection. (A, D) Splenocytes from LCMV-clone 13-infected WT, *Irf4* ^*+/fl*^ and *Irf4* ^*fl/fl*^ mice were stained with a viability dye, LCMV-specific H2-D^b^-GP276 and H2D^b^-GP33 tetramers, and antibodies to CD8, T-bet and Eomes, and analyzed between D112-114 p.i. Dot plots show CD8 versus H2-D^b^-GP276 (A) or CD8 vs H2D^b^-GP33 (D) tetramer staining on live CD8^+^ T cells. Graphs show compilations of proportions and numbers of tetramer-specific cells from D112-114 post infection. (B, E) Graphs show compilations of the numbers and proportions of T-bet^+^ Eomes^-^ H2-D^b^-GP276 (B) or H2-D^b^-GP33 (E) specific cells. (C, F) Graphs show compilations of the numbers and proportions of T-bet^hi^ PD-1^lo^ H2-D^b^-GP276 (C) or H2-D^b^-GP33 (F) specific cells. Symbols outlined in bold represent mice with undetectable titers of virus in sera at D112-114 p.i. Each data point represents an individual mouse and data are a compilation of three independent experiments; significant differences determined by Ordinary one-way ANOVA using Tukey’s multiple comparison test. $ denotes statistically significant difference between WT and *Irf4* ^*+/fl*^ samples when analyzing only mice with undetectable serum viral titers (bold outlined symbols). Significant differences between outlined samples were determined by unpaired t test with Welch’s correction.

Examination of virus-specific CD8^+^ T cells at D112-114 p.i. showed no significant differences in their numbers of virus-specific CD8^+^ T cells in *Irf4* ^*+/fl*^ mice compared to WT controls (Fig 6A and 6D). Nonetheless, consistent with our observation from D8 and D21-24 p.i., virus-specific *Irf4* ^*+/fl*^ CD8^+^ T cells at D112-114 p.i. exhibited reduced T-bet to Eomes ratio relative to WT cells (S4A and S4C Fig). This skewed ratio resulted in higher proportions and numbers of T-bet^+^ Eomes^-^ virus-specific CD8^+^ T cells in WT mice relative to *Irf4* ^*+/fl*^ mice (Fig 6B and 6E). When the data from the WT mice were segregated according to viral titers, we found that WT mice that had not controlled LCMV-clone 13 infection at this time point, appeared as outliers and had proportions and numbers of T-bet^+^ Eomes^-^ virus-specific CD8^+^ T cells comparable to *Irf4* ^*+/fl*^ mice (Fig 6B and 6E). These findings are consistent with the conclusion that persistent antigen is essential for conversion of T-bet^+^ Eomes^-^ cells into T-bet^-^ Eomes^+^ progeny [13].

Control of persistent LCMV-clone 13 infection has been associated with high numbers of T-bet^+^ virus-specific CD8^+^ T cells that lack expression of the exhaustion marker, PD-1 [13]. When analyzed at D112-114 p.i., we observed increased proportions and numbers of T-bet^hi^ PD-1^lo^ cells in WT mice relative to *Irf4* ^*+/fl*^ and *Irf4* ^*fl/fl*^ mice infected with LCMV-clone 13 (Fig 6C and 6F). However, as with the analysis of T-bet^+^ Eomes^-^ cells, the T-bet^hi^ PD-1^lo^ population in WT mice that had not cleared the virus by this timepoint appeared more similar to that of the *Irf4* ^*+/fl*^ cells. These data suggest that the proportions and numbers of T-bet^hi^ PD-1^lo^ CD8^+^ T cells may be more closely associated with viral control than with IRF4 expression levels. Similar findings are evident from analysis of Eomes^+^ PD-1^hi^ CD8^+^ T cell proportions and numbers at this timepoint (S4B and S4D Fig).

### Reducing Eomes expression improves viral control in LCMV-clone 13-infected *Irf4* ^*+/fl*^ mice

The data presented above indicated that LCMV-clone 13-infected *Irf4* ^*+/fl*^ mice have a lower T-bet to Eomes ratio than WT mice in their virus-specific CD8^+^ T cells at all time points investigated post-infection. Since optimal expression of both of these factors is essential for viral control, we considered whether manipulation of this ratio in favor of T-bet would restore protective CD8^+^ T cell responses in *Irf4* ^*+/fl*^ mice, leading to improved viral control. To address this, *Irf4* ^*+/fl*^ mice were crossed to Eomes^*+/fl*^ mice to generate *Irf4* ^*+/fl*^ Eomes^*+/fl*^ *CD4-Cre* mice (henceforth referred to as *Irf4* ^*+/fl*^ Eomes^*+/fl*^ mice). These compound heterozygotes were infected with LCMV-clone 13, along with WT, *Irf4* ^*+/fl*^, and Eomes^*+/fl*^ mice and analyzed at D77-82 p.i.

**Fig 7:**
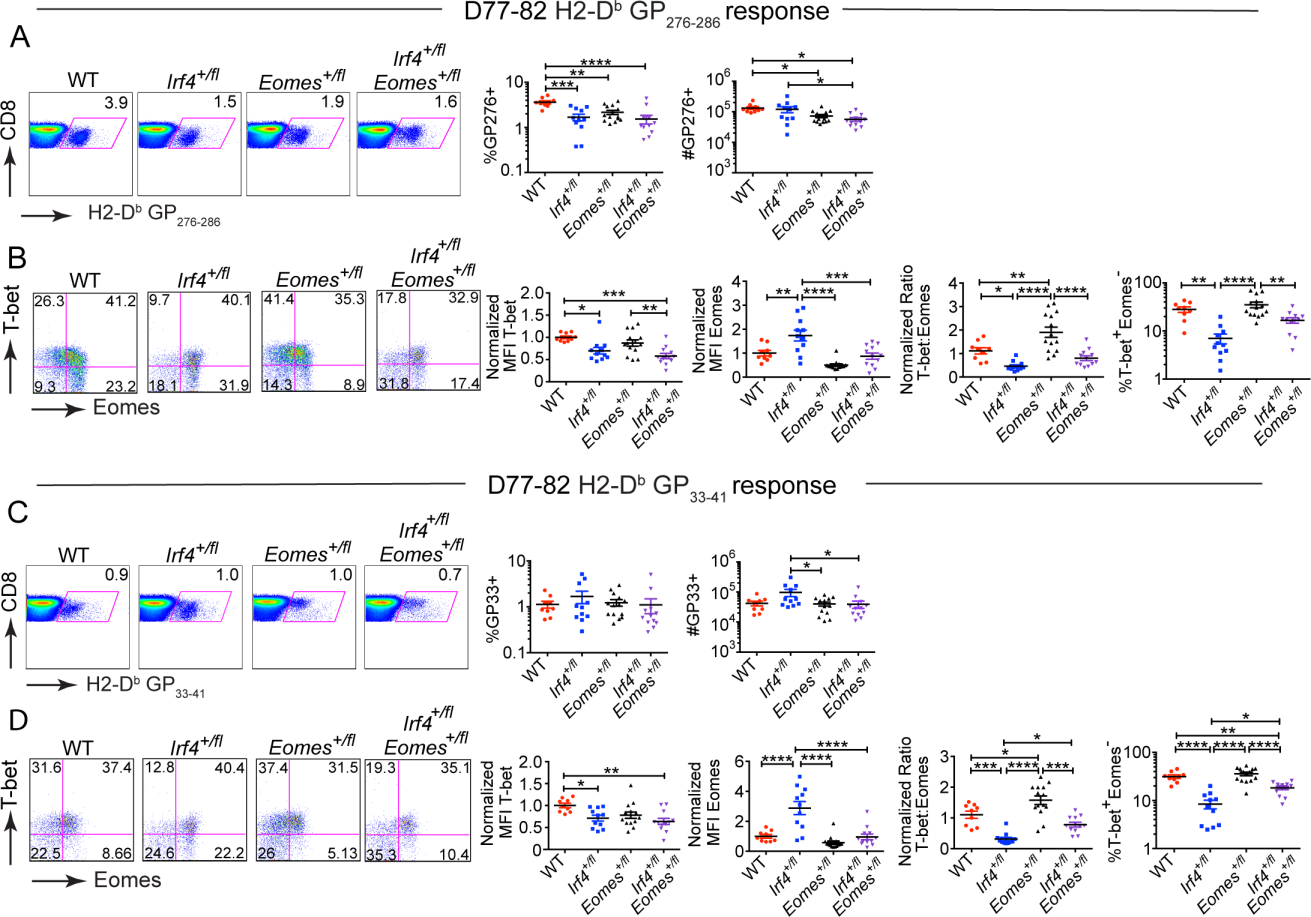
*Irf4*-*Eomes* compound haplodeficiency restores the T-bet to Eomes ratios in virus-specific CD8^+^ T cells. Splenocytes from LCMV-clone 13 infected WT, *Irf4* ^*+/fl*^, Eomes^*+/fl*^ and *Irf4* ^*+/fl*^Eomes^*+/fl*^ mice were stained with a viability dye, LCMV-specific H2-D^b^-GP276 and H2D^b^-GP33 tetramers, and antibodies to CD8, T-bet and Eomes and analyzed between D77-82 p.i. (A, C) Dot plots show CD8 vs H2-D^b^-GP276 (A) or CD8 vs H2D^b^-GP33 (C) tetramer staining on live CD8^+^ T cells. Graphs show compilations of proportions and numbers of virus-specific CD8^+^ T cells. (B, D) Dot plots show T-bet vs Eomes staining of H2-D^b^-GP276 (B) or H2-D^b^-GP33 (D) specific cells. Graphs show the MFI of T-bet and Eomes each normalized to average value for WT samples in each experiment, and the ratio of normalized MFIs for T-bet relative to Eomes, and the compilation of proportions of T-bet^+^ Eomes^-^ cells for each virus-specific subset. Each data point represents an individual mouse and data are a compilation of three independent experiments; significant differences determined by Ordinary one-way ANOVA using Tukey’s multiple comparison test

We first observed higher proportions of GP276-specific T cells in the spleens of WT mice relative to the other three genotypes. Analysis of absolute cell numbers indicated that WT mice had more GP276-specific CD8^+^ T cells than did Eomes^*+/fl*^ or *Irf4* ^*+/fl*^ Eomes^*+/fl*^ mice; in addition, *Irf4* ^*+/fl*^ Eomes^*+/fl*^ mice had significantly fewer cells than did *Irf4* ^*+/fl*^ mice (Fig 7A). In contrast, the proportions of GP33-specific CD8^+^ T cells were similar between all four genotypes with only modest differences in the absolute numbers of these virus-specific CD8^+^ T cell populations (Fig 7C).

Analysis of the levels of T-bet and Eomes in virus-specific CD8^+^ T cells between D77-82 p.i. indicated that the *Irf4* ^*+/fl*^ and the compound heterozygotes had lower T-bet expression relative to WT, as would be expected due to the role of IRF4 in positively regulating T-bet expression (Fig 7B and 7D) [15–17,30]. The expression of Eomes was higher in the *Irf4* ^*+/fl*^ cells relative to *Irf4* ^*+/fl*^ Eomes^*+/fl*^ cells, indicating that the heterozygous deficiency in Eomes was able to reduce Eomes protein expression in *Irf4* ^*+/fl*^ cells (Fig 7B and 7D). In addition, *Irf4* ^*+/fl*^ Eomes^*+/fl*^ cells showed normalized ratios of T-bet to Eomes expression levels that were comparable to those seen in WT cells. Furthermore, the re-establishment of the WT T-bet to Eomes ratio was also accompanied by increased proportions of T-bet^+^ Eomes^-^ GP33-specific cells in *Irf4* ^*+/fl*^ Eomes^*+/fl*^ mice compared to those found in *Irf4* ^*+/fl*^ mice (Fig 7D).

**Fig 8:**
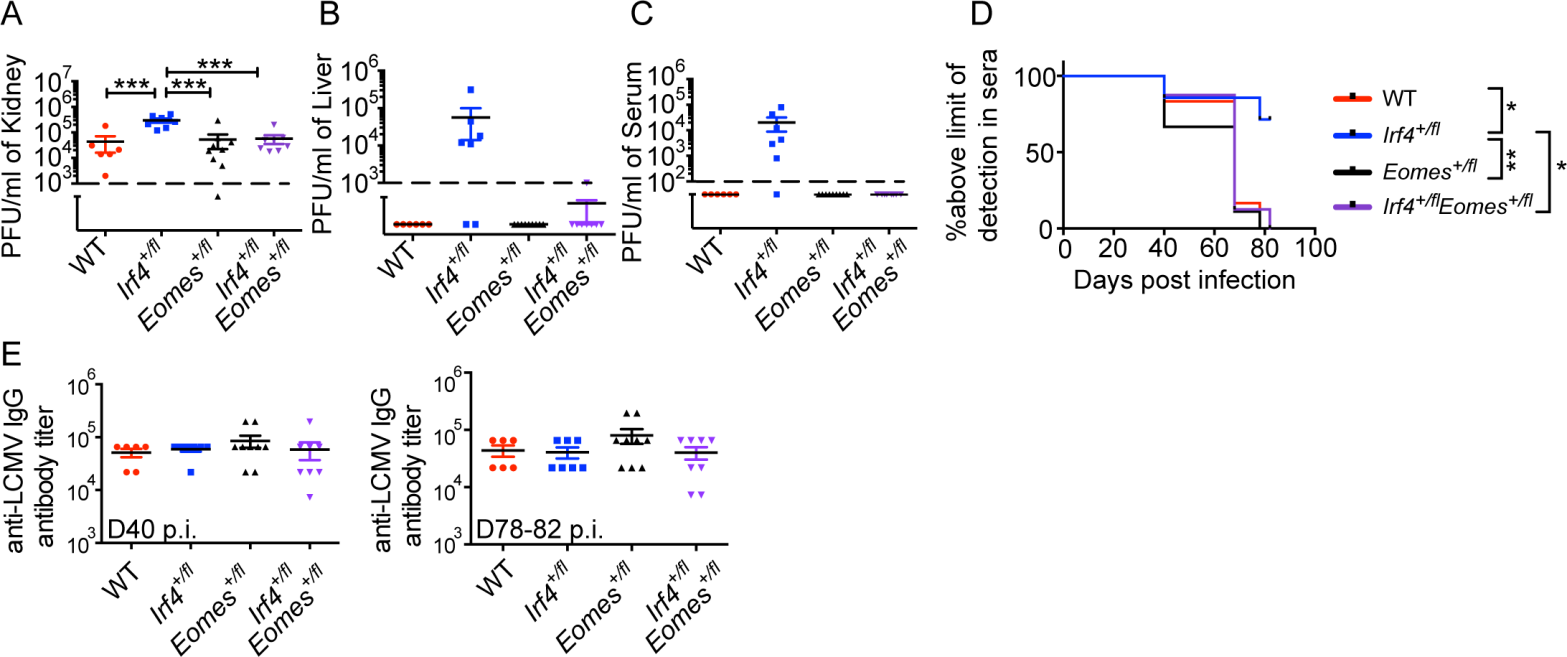
*Irf4*-*Eomes* compound haplodeficiency restores virus control during persistent LCMV-clone 13 infection. Kidney (A), livers (B) and sera (C) were harvested from LCMV-clone 13 infected WT, *Irf4* ^*+/fl*^, Eomes^*+/fl*^ and *Irf* ^*+/fl*^Eomes^*+/fl*^ mice between D77-82 post infection and virus titers were determined by plaque assay. Dotted lines indicate the limit of detection. Each data point represents an individual mouse and data are compilations of two independent experiments. (D) Serum was harvested from infected mice at various timepoints post infection. Graph indicates the proportion of mice with viral titers above the limit of detection in serum over time. Each data point represents an individual mouse and data are compilation of two independent experiments; significant differences as determined by Ordinary one-way ANOVA using Tukey’s multiple comparison test (A) and Log-rank (Mantel-Cox) test (D). E) Anti-LCMV IgG antibody titers in sera at D40 and D78-82 p.i. were determined. For D40 timepoint, data points corresponding to 3 WT and 4 *Irf4* ^*+/fl*^ mice were previously shown in Fig 5E. Each data point represents an individual mouse and data are a compilation of two independent experiments; significant differences determined by Ordinary one-way ANOVA using Tukey’s multiple comparison test.

To test the functional consequences of the restored T-bet to Eomes ratio and increased differentiation of the T-bet^+^ Eomes^-^ virus-specific CD8^+^ T cell population in *Irf4* ^*+/fl*^ Eomes^*+/fl*^ mice, we examined the viral titers in these mice between days 77–82 p.i. This analysis showed a significantly higher titer of virus in the kidneys of *Irf4* ^*+/fl*^ mice compared to the other three genotypes analyzed (Fig 8A). Additionally, in contrast to the *Irf4* ^*+/fl*^ mice, virus was efficiently cleared from livers and sera of WT, Eomes^*+/fl*^, and *Irf4* ^*+/fl*^ Eomes^*+/fl*^ mice at this timepoint (Fig 8B and 8C). Finally, we observed no difference in the kinetics of virus clearance when comparing WT to *Irf4* ^*+/fl*^ Eomes^*+/fl*^ mice (Fig 8D). These data demonstrate that reducing Eomes expression in *Irf4* ^*+/fl*^ mice restores a protective balance of T-bet to Eomes levels, leading to improved protective T cell responses to this persistent virus infection

To determine whether these mice showed differences in T cell exhaustion, we examined expression of 2B4, CD160, LAG-3, and PD-1 on virus-specific cells. At this timepoint, *Irf4* ^*fl/fl*^ cells showed a marked reduction in 2B4 expression relative to all other genotypes, and *Irf4* ^*+/fl*^ cells had higher PD-1 expression relative to WT cells (S5B Fig and S6B Fig). While WT mice exhibited higher proportions of T-bet^hi^ PD-1^lo^ and lower proportions of Eomes^hi^ PD-1^hi^ populations relative to *Irf4* ^*+/fl*^ mice, we did not observe any differences between *Irf4* ^*+/fl*^ and *Irf4* ^*+/fl*^ Eomes^*+/fl*^ cells (S5C and S5D Fig and S6C and S6D Fig). Overall, these data indicate that differences in viral control are unlikely to be due to differential expression of exhaustion markers.

We next examined cytokine production by virus-specific CD8^+^ T cells from WT, *Irf4* ^*+/fl*^, *Irf4* ^*fl/fl*^, Eomes^*+/fl*^, and *Irf4* ^*+/fl*^ Eomes^*+/fl*^ mice at D22 p.i. *Ex-vivo* stimulation of splenocytes from infected with GP276 or GP33 peptide showed few cells capable of producing IFNγ, and little evidence of multi-functional T cells in any of the mice (S5E and S5F Fig and S6E and S6F Fig). We also failed to observe a difference in Granzyme B expression between any of the genotypes, with the exception of *Irf4* ^*fl/fl*^ cells (S5H Fig and S6H Fig), arguing against a difference in cytotoxic activity between WT, *Irf4* ^*+/fl*^, Eomes^*+/fl*^, and *Irf4* ^*+/fl*^ Eomes^*+/fl*^ mice at this timepoint.

To determine whether *Irf4* ^*+/fl*^ Eomes^*+/fl*^ produced higher levels of anti-viral antibodies compared to *Irf4* ^*+/fl*^ mice, we examined anti-LCMV IgG titers at D40 and D78-82 p.i. with LCMV-clone 13. No differences in anti-viral antibody titers were observed in this analysis (Fig 8E), consistent with the comparable proportions of IL-21-producing GP61-specific CD4^+^ T cells in these mice (S6I Fig).

## Discussion

CD8^+^ T cell exhaustion is commonly observed during persistent infections and cancers [31]. Although a number of cell extrinsic factors such as the presence of the immune-suppressive cytokine IL-10, absence of help from CD4^+^ T cells, and high viral loads have been implicated in inducing exhaustion, a clear understanding of the CD8^+^ T cell intrinsic molecular mechanisms is still lacking [4,32]. The role of several transcription factors, such as Blimp-1, T-bet and Eomes, have been examined during chronic infection [13,33,34]. Blimp-1 is highly expressed in exhausted CD8^+^ T cells and its expression correlates with greater expression of exhaustion markers. Yet, *Blimp-1* knockouts have a defect in controlling LCMV-clone 13 infections relative to WT mice, whereas mice haplo-deficient for *Blimp-1* are more proficient at controlling the infection. These data indicate that varying levels of Blimp-1 regulate distinct transcriptional modules in exhausted CD8^+^ T cells, and that levels of Blimp-1 that are too high or too low are detrimental to the immune response [33]. Similarly, the expression of T-bet and Eomes segregate into T-bet^hi^ and Eomes^hi^ populations during persistent infections. Even though both proteins are T-box transcription factors and function redundantly early in LCMV Armstrong infection, loss of either protein results in impaired control of LCMV-clone 13 infections likely due to the different gene networks they regulate during LCMV-clone 13 infections [13,32]. Together these data demonstrate that efficient control of LCMV-clone 13 requires an optimal level of each of these transcription factors. Interestingly, mice lacking Blimp-1, T-bet, or Eomes have no defect in clearing acute LCMV Armstrong infections, yet are impaired in controlling LCMV-clone 13 [21,35,36]. Here we find that IRF4 joins this group of transcription factors, as reduced expression of IRF4 leads to persistence of LCMV-clone 13 infection, but does not affect clearance of LCMV Armstrong [15].

Despite the fact that the function of Tbet^hi^ and Eomes^hi^ subsets is well defined, the factors regulating the differentiation of anti-viral CD8^+^ T cells into these subsets is not known. We explored the role of TCR signaling via IRF4 in the relative expression of T-bet and Eomes and the differentiation of T-bet^+^ Eomes^-^ and T-bet^-^ Eomes^+^ subsets during chronic LCMV infection. We find that the balance of these two transcription factors is dependent on the strength of TCR signaling and reduction in the affinity or the dose of the stimulating ligand resulted in lowering of this ratio. Molecularly, the relative levels of T-bet and Eomes were dependent on the magnitude of IRF4 expression, and reduced IRF4 expression, such as those achieved by haplo-deficiency of *Irf4*, resulted in skewing of this ratio in the favor of Eomes, both *in-vitro* and *in-vivo*. Previous studies have demonstrated that IRF4, together with its binding partner, BATF, directly bind to the both the *T-bet* and *Eomes* loci in effector CD8^+^ T cells, indicating a direct role of IRF4/BATF in regulating the expression of these two genes [17,30].

The most severe consequence of the altered T-bet to Eomes ratio was the reduced differentiation of T-bet^+^ Eomes^-^ precursors, and long-term viral persistence in *Irf4* ^*+/fl*^ mice. IRF4 regulates multiple pathways such as proliferation, metabolism, and expression of effector cytokines in anti-viral CD8^+^ T cells [16,17]. Thus, it is likely that defects in any of these pathways could contribute to impaired viral clearance in *Irf4* ^*+/fl*^ mice rather than the lower T-bet to Eomes ratio. To directly test the importance of Tbet to Eomes ratio, we reduced Eomes expression in *Irf4* ^*+/fl*^ mice by generating *Irf4* ^*+/fl*^ *Eomes* ^*+/fl*^ mice. These compound heterozygotes had lower Eomes expression relative to *Irf4* ^*+/fl*^ mice, exhibited no alteration in the T-bet to Eomes ratio relative to WT cells, and showed enhanced differentiation of T-bet^+^ Eomes^-^ cells relative to *Irf4* ^*+/fl*^ mice. Consequently, *Irf4* ^*+/fl*^ *Eomes* ^*+/fl*^ mice were indistinguishable from WT mice in terms of viral clearance from multiple organs. These data, to the best of our knowledge, are the first to test the importance of relative expression of T-bet and Eomes in LCMV-clone 13 viral control.

Another consequence of reduced T-bet to Eomes ratio was the altered differentiation of T-bet^hi^ and Eomes^hi^ populations in *Irf4* ^*+/fl*^ mice. Using T-bet and Eomes knockout mice, *Paley et*. *al*. showed that both T-bet^hi^ and Eomes^hi^ subsets are important for viral control [37]. However, it is not known if reduced differentiation of one population over the other affects viral clearance. Our studies suggest that reduced differentiation of T-bet^+^ Eomes^-^ population in *Irf4* ^*+/fl*^ mice is detrimental to efficient viral control, and that increasing this population in *Irf4* ^*+/fl*^ *Eomes* ^*+/fl*^ mice restores viral control. Consistent with this observation, lower proportions of CD8^+^ T-bet^+^ Eomes^-^ cells are observed in lung transplant recipients with relapsed CMV infection relative to controllers [27]. A recent study showed that loss of FoxO1 results in higher levels of T-bet and lower levels of Eomes, thus biasing CD8^+^ T cell differentiation to the T-bet^hi^ subset. Interestingly, the *FoxO1* deficient mice were also defective in viral control [26]. Together these data underscore the importance of previous observations that both subsets of CD8^+^ T cells, the T-bet^hi^ and the Eomes^hi^ populations, play unique and essential functions during anti-viral immune responses to LCMV-clone 13 [13]. Further, these data support our conclusion that balanced differentiation of T-bet^+^ Eomes^-^ and T-bet^-^ Eomes^+^ subsets are important for viral control.

Recently, we and others have shown that the levels of IRF4 regulate the magnitude of CD8^+^ T cell expansion during acute infections [15–19]. Similar to that seen with acute infections, we find here that the levels of IRF4 also regulate the magnitude of the CD8^+^ T cell response to LCMV-clone 13 at D8 post infection. Furthermore, the increased numbers of virus-specific cells in WT mice relative to *Irf4* ^*+/fl*^ mice also resulted in a significant increase in the numbers of Tbet^+^Eomes^-^ cells at this timepoint. Somewhat surprisingly, total virus-specific CD8^+^ T cell numbers were not different between WT and *Irf4* ^*+/fl*^ mice at D22 p.i., nor were they different at later timepoints examined. Therefore, we speculate that this early difference in the magnitude of the CD8^+^ T cell response may be responsible for the ultimate ability of WT, but not Irf4^+/fl^ mice to clear LCMV clone 13.

## Materials and Methods

### Mice

Mice were bred and housed in specific pathogen-free conditions at the University of Massachusetts Medical School (UMMS) in accordance with institutional animal care and use committee guidelines. *Irf4* ^*fl/fl*^ and *Eomes* ^*fl/fl*^ mice have been described previously [38,39]. *CD4-Cre* ^*+*^ transgenic mice were a gift from Joonsoo Kang (UMMS). *Irf4* ^*fl/fl*^ mice were crossed to *CD4-Cre* ^*+*^ transgenic mice at UMMS to generate *Irf4* ^*fl/fl*^ *CD4-Cre* ^*+*^ mice. P14 TCR transgenic *TCRα* ^*-/-*^ used for *in-vitro* studies were purchased from Taconic Farms (Germantown, New York) and crossed to *Irf4* ^*fl/fl*^ *CD4-Cre* ^*+*^ mice at UMMS. P14 TCR transgenic mice used for *in-vivo* studies were a gift from Susan Kaech (Yale), and were crossed to *Irf4* ^*fl/fl*^ *CD4-cre* ^*+*^ mice to generate P14 *Irf4* ^*+/fl*^ *CD4-cre* ^*+*^ mice. *Eomes* ^*fl/fl*^ mice were purchased from The Jackson Labs (Maine) and crossed to *Irf4* ^*+/fl*^ *CD4-Cre* ^*+*^ at UMMS. *Irf4* ^*+/+*^ and *Irf4* ^*+/+*^ *CD4-Cre* ^*+*^ mice were used as WT controls.

### Virus and Infections

LCMV-clone 13 stocks were propagated in baby hamster kidney 21 cells at UMMS [40] and were generously provided by Dr. Raymond M. Welsh and amplified by us. Adult male mice (6–9 weeks old) were infected with an exhausting dose, 2X10^6^ PFU of LCMV-clone 13 i.v. For adoptive transfers, 10^4^ WT or *Irf4* ^*+/fl*^ P14 cells were i.v. transferred into WT CD4-Cre^+^ host mice one day prior to infection.

Abs, H2D^b^ monomers and staining: Eomes phycoerythrin (PE), CD244.2 (2B4) PE, T-bet PerCP-Cy5.5, Eomes PerCP-efluor® 710, CD223 (LAG-3) PerCP-eflour® 710, TNFα PerCP-efluor® 710, T-bet PE-Cy7, IFNγ eFluor® 450, IL-2 allophycocyanin (APC) CD279 (PD-1) APC were purchased from eBioscience (San Diego, CA). CD160 PE-CF594 was purchased from BD (Billerica, MA). CD8 PE-TexasRed, Granzyme B PE, LIVE/DEAD® Fixable Aqua Dead Cell Stain Kit were purchased from Life Technologies (Grand Island, NY). H2D^b^-GP33 monomers were prepared at UMMS; LCMV-specific H2D^b^-GP276 and H2D^b^-GP33 monomer were obtained from the NIH Tetramer Core Facility (Atlanta, GA). Single cell suspensions from spleens were prepared, RBC lysed and Fc receptors were blocked using supernatant from 2.4G2 hybridomas. Cells were stained with H2D^b^-GP276 or H2D^b^-GP33 tetramers prior to staining with cell-surface antibodies. For cytokine staining, cells were stimulated *ex-vivo* with 1μg/ml GP_276-286_ or GP_33-41_ peptide for 5 hours at 37°C. Intracellular cytokine staining was performed using BD Cytofix/Cytoperm™ Fixation/Permeabilization Solution kit. Intracellular transcription factor staining was performed using eBioscience Foxp3/transcription factor staining kit, as per manufacturer’s instructions, unless specified. All samples were analyzed on an LSRII flow cytometer (BD Biosciences), and data were analyzed using FlowJo (Tree Star).

### Peptides

LCMV peptides GP_276-286_ (SGVENPGGYCL), GP_33-41_ (KAVYNFATC) and F6L (KAVYNLATC) were synthesized and HPLC purified by 21^st^ century Biochemicals (Marlboro, MA). Peptides were ~90% pure.

### Cell culture

Lymph node cells from P14 WT, P14 *Irf4* ^*+/fl*^ or P14 *Irf4* ^*fl/fl*^ mice were mixed with equal numbers of WT CD45.1 splenocytes and stimulated with GP_33–41_ (GP33) or F6L peptides for 24, 48, and 72 h. Cells were harvested and analyzed for T-bet and Eomes expression by intracellular staining.

### Viral Titers

Sera, livers and kidneys were harvested from infected mice at the indicated time points post-infection (p.i.). Organs were homogenized in one ml of complete RPMI media and stored at -80°C. LCMV-clone 13 virus titers were determined by plaque assays as previously described [40].

### LCMV-Specific Antibody Titers

To quantify LCMV-specific antibody titers, high-binding 96 well flat bottom ELISA plates (Corning) were coated overnight at room temperature with cell lysate from LCMV-clone 13 infected BHK21 cells. Plates were washed, blocked and three-fold serial dilutions of serum samples were plated. Plates were washed again and incubated with horseradish peroxidase labeled goat anti-mouse IgG detection antibody (Bethyl labs) and developed using 3,3',5,5'-Tetramethylbenzidine substrate. The reaction was quenched 0.18M H_2_SO_4_ ELISA stop solution (Bethyl Labs) and the optical density (OD) was measured at 450nm using an Emax Endpoint ELISA microplate reader (Molecular Devices). LCMV-specific antibody titers were determined by end-point titer method and two times the mean OD of uninfected control sera was used as the cut-off.

### Statistical Analysis

All data are represented as mean ± Standard Error of Mean (SEM). Statistical significance is indicated by ns *p*>0.05, *p≤0.05, **p≤0.01, ***p≤0.001, and ****p≤0.0001. For outlined D112-114 samples, statistical significance is indicated by $*p*≤0.05 and $ $*p*≤0.01. Statistical analysis was performed using unpaired t test with Welch’s correction, ordinary one-way ANOVA using Tukey’s multiple comparison test or Log-rank (Mantel-Cox) test as indicated in the figure legend.

## Supporting Information

S1 Fig IRF4 regulates T-bet and Eomesodermin levels in stimulated CD8^+^ T cells. P14 WT, P14 *Irf4* ^*+/fl*^ or P14 *Irf4* ^*fl/fl*^ cells were stimulated with the indicated concentrations of GP33 and F6L peptides *in-vitro*. At 24, 48, and 72 h, cells were stained and analyzed for T-bet expression. The graphs show the raw MFIs for T-bet at each time-point. Data were generated from gated live CD8^+^CD45.2^+^CD44^hi^ T cells analyzed in four independent experiments.
(TIF)

S2 Fig *Irf4* gene dosage regulates CD8^+^ T cell clonal expansion in response to LCMV-clone 13 infection as well as the differentiation of T-bet^hi^ and Eomes^hi^ subsets. (A) Splenocytes from LCMV-clone 13-infected WT, *Irf4* ^*+/fl*^ and *Irf4* ^*fl/fl*^ mice were harvested at D8 p.i. and stained with a viability dye, LCMV-specific H2D^b^-GP33 tetramer, and antibodies to CD8, T-bet and Eomes. Dot plots show CD8 versus H2-D^b^-GP33 tetramer staining. Graphs show compilations of proportions and numbers from D8 post-infection (right). (B) Representative dot plots show T-bet vs Eomes staining on gated CD8^+^ live H2-D^b^-GP33 specific cells at D8 p.i. Graphs show the MFI of T-bet and Eomes each normalized to the average of WT samples in each experiment for live CD8^+^ H2-D^b^-GP33 specific cells, and the ratio of normalized MFIs for T-bet relative to Eomes. (C) Graphs show compilations of proportions and numbers of T-bet^+^ Eomes^-^ and T-bet^+^ Eomes^+^ cells. Each data point represents an individual mouse and data are a compilation of three independent experiments; significant differences determined by Ordinary one-way ANOVA using Tukey’s multiple comparison test.
(TIF)

S3 Fig *Irf4* gene dosage regulates the proportions of virus-specific CD8^+^ T cells during persistent LCMV-clone 13 infection. Splenocytes from LCMV-clone 13 infected WT, *Irf4* ^*+/fl*^ and *Irf4* ^*fl/fl*^ mice were harvested between D21-24 p.i. and stained with a viability dye, LCMV-specific H2-D^b^-GP276 and H2D^b^-GP33 tetramers, and antibodies to CD8, T-bet and Eomes. (A, C) Graphs show the numbers and proportions of T-bet^+^ Eomes^+^ (left) and T-bet^-^ Eomes^+^ (right) populations. Each data point represents an individual mouse and data are compilations of five independent experiments; significant differences determined by Ordinary one-way ANOVA using Tukey’s multiple comparison test. (B, D) Dot plots of uninfected control and LCMV Armstrong infected control used to determine gating of T-bet versus Eomes for each tetramer stained subset.
(TIF)

S4 Fig Clearance of LCMV-clone 13 leads to increased T-bet to Eomesodermin ratios. Splenocytes from LCMV-clone 13-infected WT, *Irf4* ^*+/fl*^ and *Irf4* ^*fl/fl*^ mice were stained with a viability dye, LCMV-specific H2-D^b^-GP276 and H2D^b^-GP33 tetramers, and antibodies to CD8, T-bet and Eomes, and analyzed between D112-114 p.i. Graphs show the MFI of T-bet and Eomes each normalized to the average of WT samples, and the ratio of normalized MFIs for T-bet relative to Eomes, for live CD8^+^ H2-D^b^-GP276 (A) and H2-D^b^-GP33 (C) specific cells. Graphs show compilations of the numbers and proportions of Eomes^hi^ PD-1^hi^ H2-D^b^-GP276 (B) or H2-D^b^-GP33 (D) specific cells. Each data point represents an individual mouse and data are a compilation of three independent experiments; significant differences determined by Ordinary one-way ANOVA using Tukey’s multiple comparison test. Symbols with bold outlines represent mice whose serum viral titers were below the limit of detection at D112-114 p.i.. $ denotes statistically significant difference between WT and *Irf4* ^*+/fl*^ samples when analyzing only mice with undetectable serum viral titers (bold outlined symbols). Significant differences between bold outlined samples were determined by unpaired t test with Welch’s correction.
(TIF)

S5 Fig Compound haplo-deficiency of *Irf4* and *Eomes* does not alter exhaustion marker expression, cytokine production, or effector function in H2-D^b^-GP276 specific cells. Splenocytes from LCMV-clone 13-infected WT, *Irf4* ^*+/fl*^, *Irf4* ^*fl/fl*^, *Eomes* ^*+/fl*^ and *Irf4* ^*+/fl*^ *Eomes* ^*+/fl*^ mice were stained with a viability dye, LCMV-specific H2-D^b^-GP276 tetramers, and antibodies to CD8, T-bet, Eomes, 2B4, CD160, LAG-3, PD-1, and granzyme B and analyzed at D22 p.i. (A) Number of H2-D^b^-GP276 specific cells at D22 p.i. (B) Graphs show the proportions of 2B4-, CD160-, LAG-3-, and PD-1-positive H2-D^b^-GP276 specific cells at D22 p.i. (C) Dot plots show T-bet versus PD-1 staining on H2-D^b^-GP276 specific CD8^+^, live cells. Graph shows the proportions of T-bet^hi^ PD-1^lo^ H2-D^b^-GP276 CD8^+^ specific cells. * Indicates statistically significant differences relative to WT samples. (D) Dot plots show Eomes versus PD-1 staining on H2-D^b^-GP276 specific, CD8^+^, live cells. Graph shows proportions of Eomes^hi^ PD-1^hi^ H2-D^b^-GP276 CD8^+^ specific cells. (E-H) Splenocytes from LCMV-clone 13-infected WT, *Irf4* ^*+/fl*^, *Irf4* ^*fl/fl*^, *Eomes* ^*+/fl*^ and *Irf4* ^*+/fl*^ *Eomes* ^*+/fl*^ mice were isolated at D22 p.i. and stimulated with GP276 peptide, stained with a viability dye and antibodies to CD8, IFNγ, TNFα and IL-2. (E) Dot plots show representative staining of WT CD8^+^ live cells (CD8 versus IFNγ) and gated IFNγ^+^ CD8^+^ live cells (TNFα versus IL-2). (F) Graph shows the proportions of IFNγ^+^ cells gated on CD8^+^ live cells for each genotype. (G) Graphs show the proportions of TNFα^+^ IL-2^-^ (left) and TNFα^+^ IL-2^+^ (right) cells gated on IFNγ^+^ CD8^+^ live cells for each genotype. (H) Graph shows the numbers of Granzyme B^+^ H2-D^b^-GP276 CD8^+^ live cells for each genotype. Each data point represents an individual mouse and data are compilations of three independent experiments; significant differences were determined by Ordinary one-way ANOVA using Tukey’s multiple comparison test.
(TIF)

S6 Fig Compound haplo-deficiency of *Irf4* and *Eomes* does not alter exhaustion marker expression, cytokine production, or effector function in H2-D^b^-GP33 specific cells. Splenocytes from LCMV-clone 13-infected WT, *Irf4* ^*+/fl*^, *Irf4* ^*fl/fl*^, *Eomes* ^*+/fl*^ and *Irf4* ^*+/fl*^ *Eomes* ^*+/fl*^ mice were stained with a viability dye, LCMV-specific H2-D^b^-GP33 tetramers, and antibodies to CD8, T-bet, Eomes, 2B4, CD160, LAG-3, PD-1, and granzyme B and analyzed at D22 p.i. (A) Number of H2-D^b^-GP33 specific cells at D22 p.i. (B) Graphs show the proportions of 2B4-, CD160-, LAG-3-, and PD-1-positive H2-D^b^-GP33 specific cells at D22 p.i. (C) Dot plots show T-bet versus PD-1 staining on H2-D^b^-GP33 specific, CD8^+^, live cells. Graph shows the proportions of T-bet^hi^ PD-1^lo^ H2-D^b^-GP33 CD8^+^ specific cells. * Indicates statistically significant differences relative to WT samples. (D) Dot plots show Eomes versus PD-1 staining on H2-D^b^-GP33 specific, CD8^+^, live cells. Graph shows proportions of Eomes^hi^ PD-1^hi^ H2-D^b^-GP33 CD8^+^ specific cells. (E-H) Splenocytes from LCMV-clone 13-infected WT, *Irf4* ^*+/fl*^, *Irf4* ^*fl/fl*^, *Eomes* ^*+/fl*^ and *Irf4* ^*+/fl*^ *Eomes* ^*+/fl*^ mice were isolated at D22 p.i. and stimulated with GP33 peptide, stained with a viability dye and antibodies to CD8, IFNγ, TNFα, and IL-2. (E) Dot plots show representative staining of WT CD8^+^ live cells (CD8 versus IFNγ) and gated IFNγ^+^ CD8^+^ live cells (TNFα versus IL-2). (F) Graph shows the proportions of IFNγ^+^ cells gated on CD8^+^ live cells for each genotype. (G) Graphs show the proportions of TNFα^+^ IL-2^-^ (left) and TNFα^+^ IL-2^+^ (right) cells gated on IFNγ^+^ CD8^+^ live cells for each genotype. (H) Graph shows the numbers of Granzyme B^+^ H2-D^b^-GP33 specific CD8^+^ live cells for each genotype. Each data point represents an individual mouse and data are compilations of two (A-D,H-I) or three (E-G) independent experiments; significant differences were determined by Ordinary one-way ANOVA using Tukey’s multiple comparison test.
(TIF)
