# Supplementary figures and images for: IRF4 Regulates the Ratio of T-Bet to Eomesodermin in CD8+ T Cells Responding to Persistent LCMV Infection
Source: PLoS One. 2015 Dec 29;10(12):e0144826. doi: 10.1371/journal.pone.0144826 (PMC4699851; doi:10.1371/journal.pone.0144826)

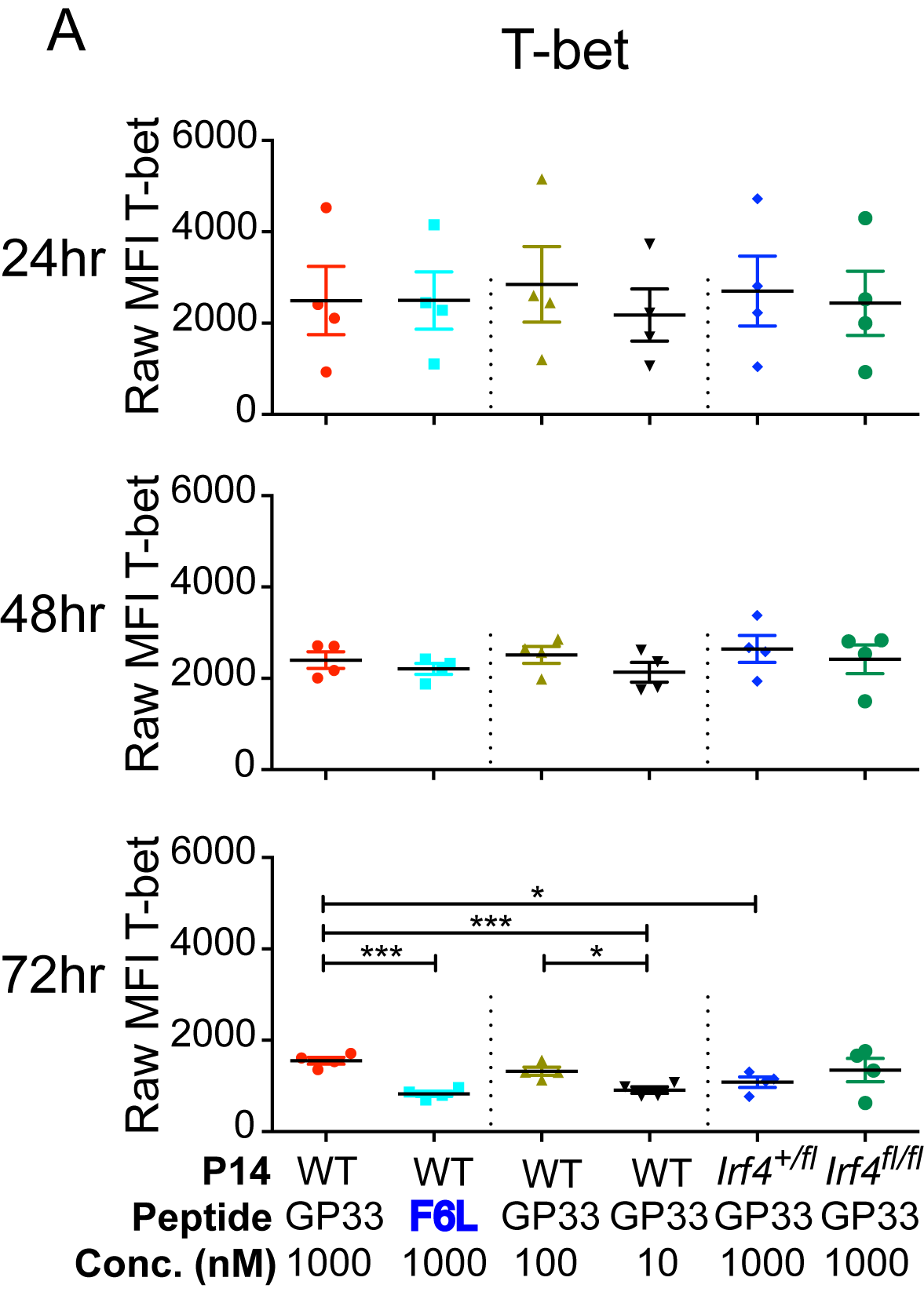

Supplement: S1 Fig — P14 WT, P14 Irf4 +/fl or P14 Irf4 fl/fl cells were stimulated with the indicated concentrations of GP33 and F6L peptides in-vitro. At 24, 48, and 72 h, cells were stained and analyzed for T-bet expression. The graphs show the raw MFIs for T-bet at each time-point. Data were generated from gated live CD8+CD45.2+CD44hi T cells analyzed in four independent experiments. (TIF) [file pone.0144826.s001.tif]

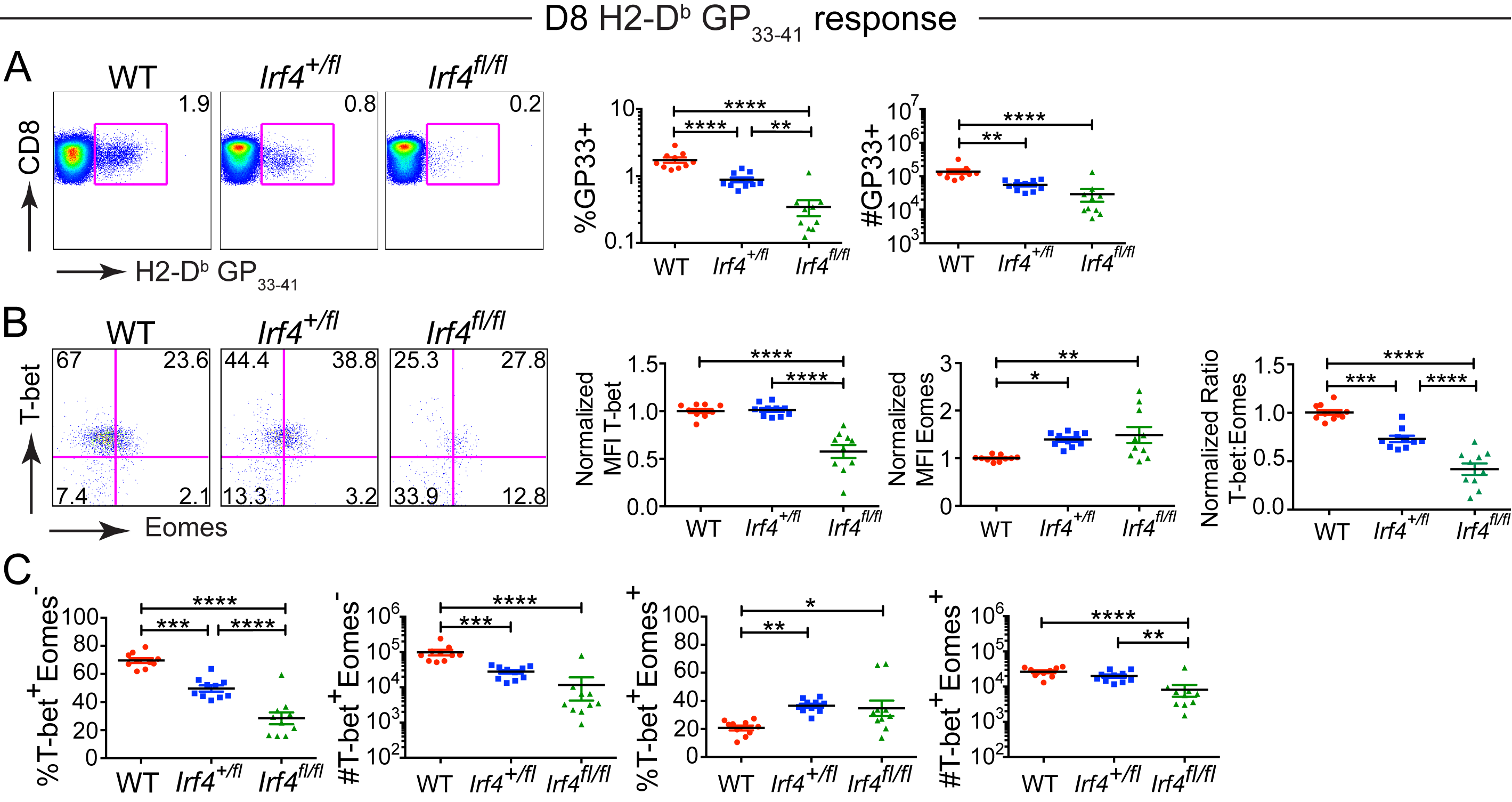

Supplement: S2 Fig — (A) Splenocytes from LCMV-clone 13-infected WT, Irf4 +/fl and Irf4 fl/fl mice were harvested at D8 p.i. and stained with a viability dye, LCMV-specific H2Db-GP33 tetramer, and antibodies to CD8, T-bet and Eomes. Dot plots show CD8 versus H2-Db-GP33 tetramer staining. Graphs show compilations of proportions and numbers from D8 post-infection (right). (B) Representative dot plots show T-bet vs Eomes staining on gated CD8+ live H2-Db-GP33 specific cells at D8 p.i. Graphs show the MFI of T-bet and Eomes each normalized to the average of WT samples in each experiment for live CD8+ H2-Db-GP33 specific cells, and the ratio of normalized MFIs for T-bet relative to Eomes. (C) Graphs show compilations of proportions and numbers of T-bet+ Eomes- and T-bet+ Eomes+ cells. Each data point represents an individual mouse and data are a compilation of three independent experiments; significant differences determined by Ordinary one-way ANOVA using Tukey’s multiple comparison test. (TIF) [file pone.0144826.s002.tif]

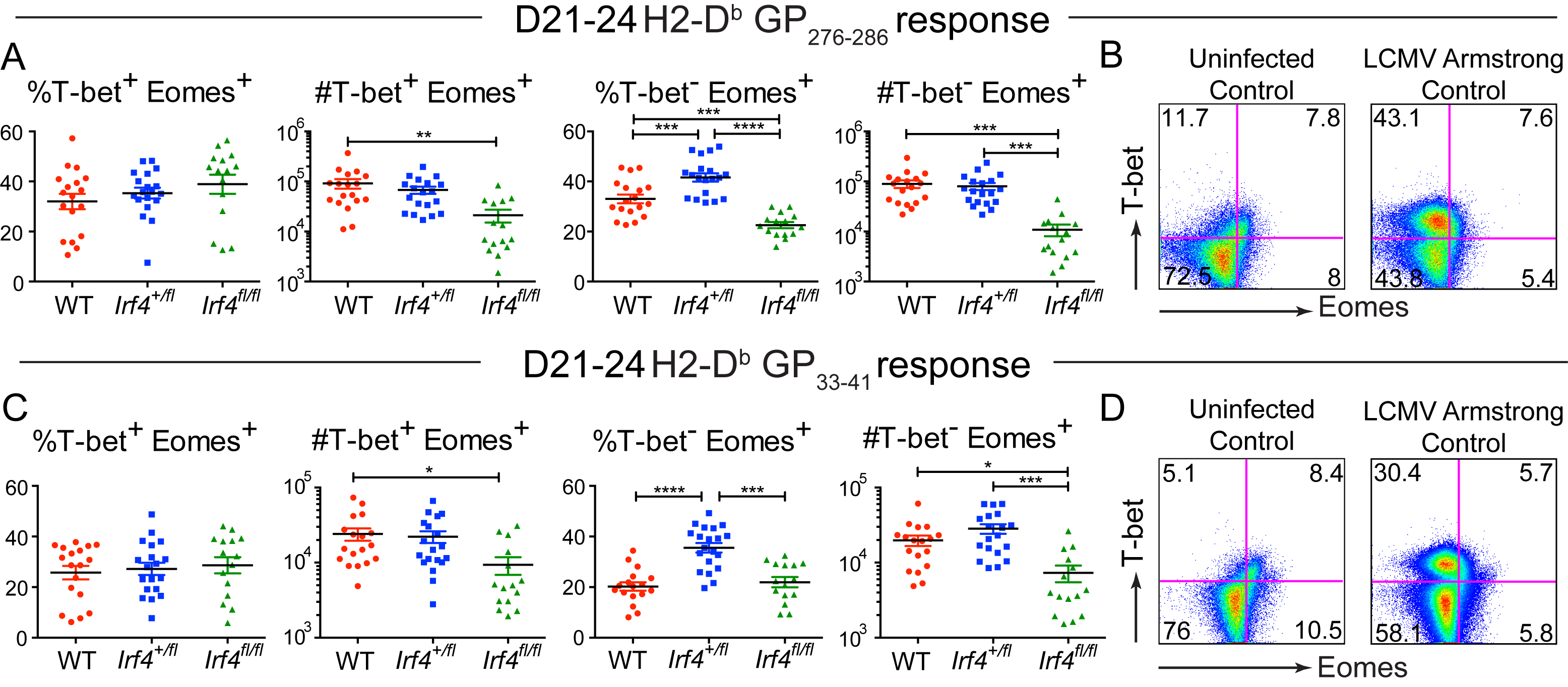

Supplement: S3 Fig — Splenocytes from LCMV-clone 13 infected WT, Irf4 +/fl and Irf4 fl/fl mice were harvested between D21-24 p.i. and stained with a viability dye, LCMV-specific H2-Db-GP276 and H2Db-GP33 tetramers, and antibodies to CD8, T-bet and Eomes. (A, C) Graphs show the numbers and proportions of T-bet+ Eomes+ (left) and T-bet- Eomes+ (right) populations. Each data point represents an individual mouse and data are compilations of five independent experiments; significant differences determined by Ordinary one-way ANOVA using Tukey’s multiple comparison test. (B, D) Dot plots of uninfected control and LCMV Armstrong infected control used to determine gating of T-bet versus Eomes for each tetramer stained subset. (TIF) [file pone.0144826.s003.tif]

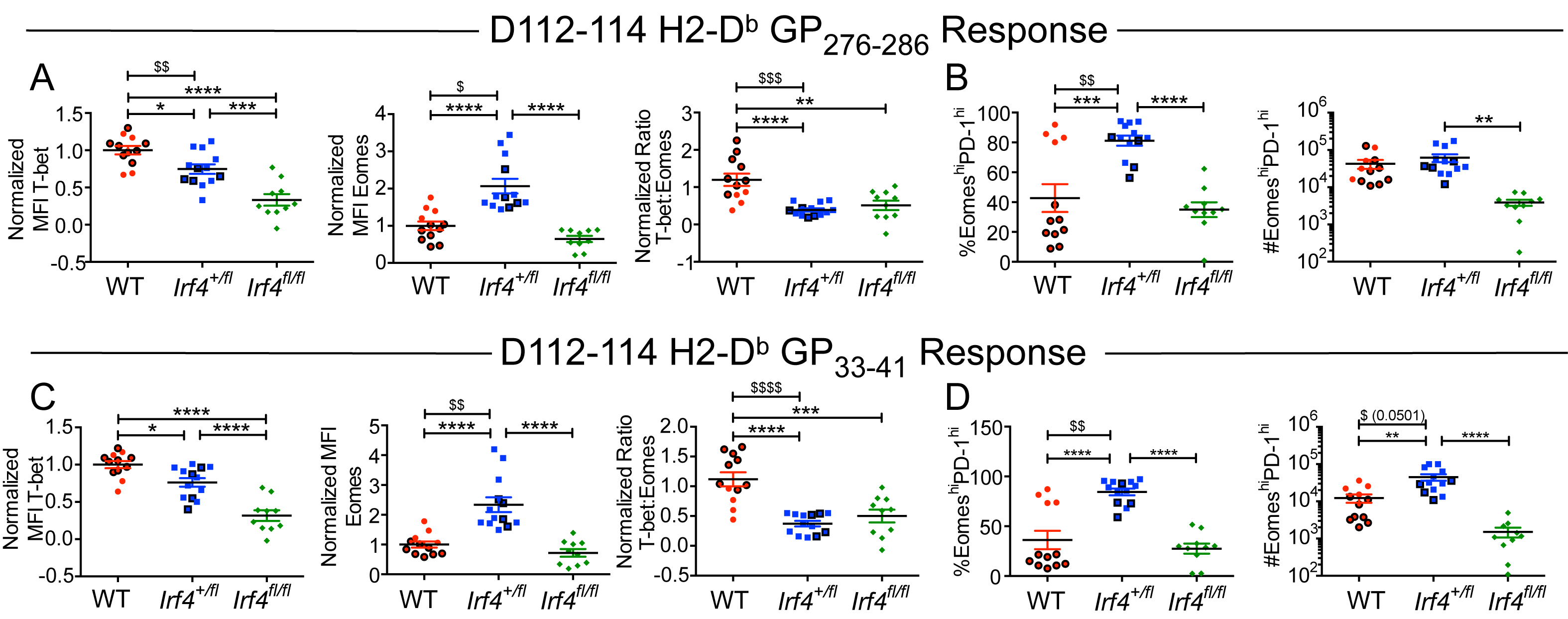

Supplement: S4 Fig — Splenocytes from LCMV-clone 13-infected WT, Irf4 +/fl and Irf4 fl/fl mice were stained with a viability dye, LCMV-specific H2-Db-GP276 and H2Db-GP33 tetramers, and antibodies to CD8, T-bet and Eomes, and analyzed between D112-114 p.i. Graphs show the MFI of T-bet and Eomes each normalized to the average of WT samples, and the ratio of normalized MFIs for T-bet relative to Eomes, for live CD8+ H2-Db-GP276 (A) and H2-Db-GP33 (C) specific cells. Graphs show compilations of the numbers and proportions of Eomeshi PD-1hi H2-Db-GP276 (B) or H2-Db-GP33 (D) specific cells. Each data point represents an individual mouse and data are a compilation of three independent experiments; significant differences determined by Ordinary one-way ANOVA using Tukey’s multiple comparison test. Symbols with bold outlines represent mice whose serum viral titers were below the limit of detection at D112-114 p.i.. $ denotes statistically significant difference between WT and Irf4 +/fl samples when analyzing only mice with undetectable serum viral titers (bold outlined symbols). Significant differences between bold outlined samples were determined by unpaired t test with Welch’s correction. (TIF) [file pone.0144826.s004.tif]

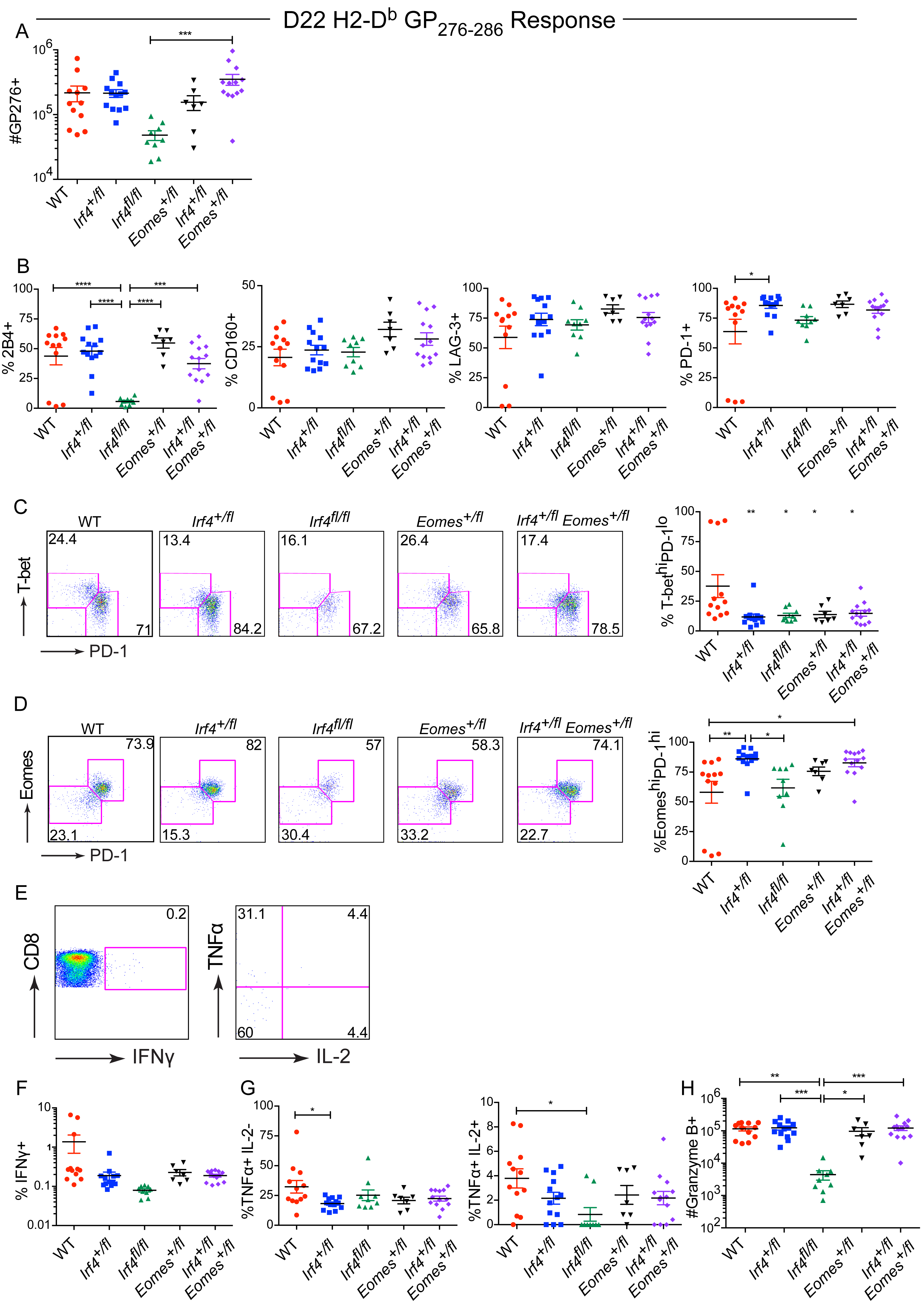

Supplement: S5 Fig — Splenocytes from LCMV-clone 13-infected WT, Irf4 +/fl, Irf4 fl/fl, Eomes +/fl and Irf4 +/fl Eomes +/fl mice were stained with a viability dye, LCMV-specific H2-Db-GP276 tetramers, and antibodies to CD8, T-bet, Eomes, 2B4, CD160, LAG-3, PD-1, and granzyme B and analyzed at D22 p.i. (A) Number of H2-Db-GP276 specific cells at D22 p.i. (B) Graphs show the proportions of 2B4-, CD160-, LAG-3-, and PD-1-positive H2-Db-GP276 specific cells at D22 p.i. (C) Dot plots show T-bet versus PD-1 staining on H2-Db-GP276 specific CD8+, live cells. Graph shows the proportions of T-bethi PD-1lo H2-Db-GP276 CD8+ specific cells. * Indicates statistically significant differences relative to WT samples. (D) Dot plots show Eomes versus PD-1 staining on H2-Db-GP276 specific, CD8+, live cells. Graph shows proportions of Eomeshi PD-1hi H2-Db-GP276 CD8+ specific cells. (E-H) Splenocytes from LCMV-clone 13-infected WT, Irf4 +/fl, Irf4 fl/fl, Eomes +/fl and Irf4 +/fl Eomes +/fl mice were isolated at D22 p.i. and stimulated with GP276 peptide, stained with a viability dye and antibodies to CD8, IFNγ, TNFα and IL-2. (E) Dot plots show representative staining of WT CD8+ live cells (CD8 versus IFNγ) and gated IFNγ+ CD8+ live cells (TNFα versus IL-2). (F) Graph shows the proportions of IFNγ+ cells gated on CD8+ live cells for each genotype. (G) Graphs show the proportions of TNFα+ IL-2- (left) and TNFα+ IL-2+ (right) cells gated on IFNγ+ CD8+ live cells for each genotype. (H) Graph shows the numbers of Granzyme B+ H2-Db-GP276 CD8+ live cells for each genotype. Each data point represents an individual mouse and data are compilations of three independent experiments; significant differences were determined by Ordinary one-way ANOVA using Tukey’s multiple comparison test. (TIF) [file pone.0144826.s005.tif]
